# Variation in how cognitive control modulates sentence processing

**DOI:** 10.1098/rsos.211969

**Published:** 2023-04-19

**Authors:** Abhijeet Patra, Jeremy Kirkwood, Erica L. Middleton, Malathi Thothathiri

**Affiliations:** ^1^ Moss Rehabilitation Research Institute, Elkins Park, PA, USA; ^2^ Faculty of Health and Education, Manchester Metropolitan University, Manchester, UK; ^3^ Department of Speech, Language and Hearing Sciences, The George Washington University, Washington, DC 20052, USA

**Keywords:** executive function, syntax, conflict adaptation, conflict resolution, sentence comprehension, individual differences

## Abstract

Prior research suggests that cognitive control can assist the comprehension of sentences that create conflict between interpretations, at least under some circumstances. However, the mixed pattern of results suggests that cognitive control may not always be necessary for accurate comprehension. We tested whether cognitive control recruitment for language processing is systematically variable, depending on the type of sentential ambiguity or conflict, individual differences in cognitive control, and task demands. Participants completed two sessions in a web-based experiment. The first session tested conflict modulation using interleaved Stroop and sentence comprehension trials. Critical sentences contained syntax-semantics or phrase-attachment conflict. In the second session, participants completed three cognitive control and three working memory tasks. Exploratory factor analysis was used to index individual differences in a cognitive control factor and a working memory factor. At the group level, there were no significant conflict modulation effects for either syntax-semantics or phrase-attachment conflict. At the individual differences level, the cognitive control factor correlated with offline comprehension accuracy but not online processing measures for both types of conflict. Together, the results suggest that the role of cognitive control in sentence processing may vary according to task demands. When overt decisions are required, individual differences in cognitive control may matter such that better cognitive control results in better language comprehension performance. The results add to the mixed evidence on conflict modulation and raise questions about the situations under which cognitive control influences online processing.

## 1. Introduction

Language comprehension unfolds quickly from moment to moment. We do not wait to read or hear the ends of words and sentences before interpreting them. This incremental processing is efficient but can lead to comprehension difficulty when there is a mismatch between what the brain expects to come next and what actually comes next. For example, the comprehension of an ambiguous ‘garden-path’ sentence like ‘Put the apple on the towel in the box’ shows evidence for the temporary misinterpretation of ‘on the towel’ as a destination (where to put the apple), which must be revised to correctly interpret ‘on the towel’ as a modifier, i.e. which apple should be moved [1]. Such hitches during moment-to-moment comprehension can be detected using eye movement patterns and slowed reaction times at specific time points during a sentence [1–3].

Because language routinely contains interpretive ambiguities, the comprehension system must possess one or more mechanisms for arriving at the correct interpretation after an initial misinterpretation. Following Novick *et al.* [4], there is growing interest in whether cognitive control might be one such mechanism. Cognitive control refers to the broad ability to regulate behaviour in accordance with the current context and goals. For example, in the commonly used Stroop task, participants must name the font colour of written colour words. Responding ‘blue’ to the word ‘orange’ written in blue font requires cognitive control because participants must inhibit their default tendency to read the word aloud and instead produce the response corresponding to the font colour. Novick *et al*. [4] suggested that this ability—to inhibit a prepotent representation or response and choose a different representation or response—might play a role in language comprehension. Specifically, it can support the inhibition of a prepotent interpretation and the selection of an alternative meaning. Evidence in favour of this proposal has grown during the last two decades but the results are mixed and there remain important open questions about whether cognitive control is strictly necessary for revision or whether it plays a facilitative role under some situations only [5–8]. Below, we first review the evidence and the range of theoretical claims before articulating the hypotheses tested in the present study.

A number of earlier studies reported co-occurring cognitive control and sentence comprehension deficits in patients with aphasia, and the involvement of the same frontal regions in both functions in healthy adults, suggesting a link. For example, Novick *et al*. [9] reported that a patient with deficits in conflict resolution tasks also showed difficulty in producing and comprehending language, specifically in situations with competing representations. Vuong & Martin [10] found that two patients with left frontal cortex damage and cognitive control deficits performed worse on the comprehension of sentences that involved overcoming conflict with a prior bias or interpretation, relative to a patient with non-frontal damage. In healthy adults, frontal regions engaged during cognitive control tasks are also recruited for the comprehension of sentences with conflicting cues or interpretations [11–14].

More recently, this correlational evidence has been augmented by causal evidence from a conflict modulation paradigm. In this paradigm, a cognitive control task (e.g. Stroop) is interleaved with a sentence comprehension task to see if the engagement of cognitive control during the Stroop trial modulates the processing of a subsequent sentence trial. Multiple recent studies have found evidence in favour of such a causal relationship [15–17]. For example, Thothathiri *et al*. [16] reported that a prior incongruent Stroop trial facilitated the resolution of conflicting semantic and syntactic cues during the interpretation of sentences like ‘The fox was chased by the rabbit’, where the meaning indicated by syntax contradicts real-world semantic knowledge (typically, foxes chase rabbits rather than vice versa). Similarly, Hsu & Novick [15] found an effect of the previous Stroop trial on looks and actions in response to sentences like ‘Put the frog on the napkin onto the box’. Together, the conflict modulation evidence suggests that processes triggered during a non-sentence-based cognitive control task can causally influence the online processing of sentences, at least under some situations.

On the flip side, other studies have failed to find reliable correlational or causal links between cognitive control and sentence processing. In a large lesion-symptom mapping study, Thothathiri *et al*. [7] did not find an association between left frontal cortex damage and impairment in sentence comprehension. The authors concluded that frontally mediated cognitive control functions might not always be used during comprehension. Echoing this, contrastive case studies in Thothathiri & Mauro [8] showed that the patients' conflict resolution performance did not predict sentence comprehension accuracy. Using the conflict modulation paradigm, Kaan *et al.* found that the processing of reduced relative clause ambiguities was not reliably facilitated by incongruent Stroop or Flanker trials [18,19]. The authors concluded that the previous positive effects of conflict modulation (e.g. [15,16]) do not necessarily generalize to other ambiguities.

Overall, the positive evidence for a link between cognitive control and comprehension has led to a broad consensus that cognitive control is at least sometimes engaged during language processing (e.g. [6]). But the mixed pattern of results suggests that it is not always engaged, and it might not be necessary for accurate comprehension. We view cognitive control recruitment for language processing as flexible such that different individuals may or may not engage these mechanisms depending on their cognitive control ability, the type of sentence, or task demands. Under this perspective, the question shifts from a dichotomy (cognitive control is useful for language comprehension versus not) to a more nuanced question about when cognitive control is engaged and when it is not. The present study represents an attempt to answer this question. We hypothesized that cognitive control engagement during sentence comprehension may vary systematically depending on (i) the type of sentential ambiguity, (ii) the individual, and (iii) task demands. We discuss each of these possibilities separately below before summarizing the design and hypotheses of the study.

### 1.1. Type of sentential ambiguity or conflict

The hypothesis we investigated is ‘conflict-specific’ because we propose that cognitive control will specifically assist in resolving conflict between cues and interpretations during sentence comprehension. Sentence processing may be subject to different types of conflict, however. Consider two kinds of conflict-inducing sentences (1–2) that previous studies have already investigated using the conflict modulation paradigm [15,16]:

(1) Syntax-semantics conflict (hereafter, SS): *The fox was chased by the rabbit* (cf. no-conflict: *The rabbit was chased by the fox*)
(2) Phrase-attachment conflict (hereafter, PA): *Put the frog on the napkin onto the box* (cf. no-conflict: *Put the frog that's on the napkin onto the box*)

In (1), conflict arises from the need to suppress the interpretation suggested by semantics (which animal usually chases which animal) and choose the interpretation indicated by syntax (hence, syntax-semantics conflict). A plethora of previous evidence suggests that semantics, including knowledge about the plausibility of an event, can influence sentence processing ([20–22]; *inter alia*). Therefore, in cases where the syntax-guided interpretation deviates from what is semantically plausible, cognitive control could potentially help resolve the mismatch. Neuroimaging evidence has led several researchers to propose that sentence processing may proceed along multiple streams that can operate independently and in parallel (e.g. [23–30]). While the precise nature of the streams varies between models, a common thread is the differentiation between a semantic or pragmatic stream that arrives at sentence interpretation using semantic or real-world plausibility information, and a combinatorial stream that relies on combinatorial or morphosyntactic information. When the outputs of these parallel streams conflict, cognitive control could be used to monitor and resolve that conflict (e.g. [29]). This cross-stream function is consistent with a role for the frontal cortex in adjudicating between disparate sources of information in a variety of situations (e.g. a word reading stream and a visual colour processing stream in the Stroop task).^1^

By contrast to (1), the conflict in (2) arises from the violation of syntactic expectations. Sentences with the verb ‘put’ obligatorily specify a destination for the put action. Thus, ‘on the napkin’ is likely to be interpreted initially as a destination. The syntactic processor might expect the sentence to end there (Put the frog on the napkin), or continue with adjunctive information (e.g. an adverb like ‘gently’) or a conjunction (e.g. ‘and then…’). Critically, the parser is unlikely to expect another prepositional phrase such as ‘onto the box’, which indicates the actual destination. When the syntactic parser encounters the disambiguating second prepositional phrase, it must syntactically reanalyse the sentence such that ‘on the napkin’ is a modifier attached to the noun (which frog is to be acted upon) instead of a destination attached to the verb (where to put the frog). In these instances, there can be conflict between an initial syntactic representation and a subsequent syntactic representation (or correspondingly, the initial and subsequent semantic representations related to those parses), which can be resolved using cognitive control. Importantly, however, such conflict could potentially and alternatively also be handled by revision within the syntactic processing stream, which is likely to be highly practised in structure building and re-building.

As reviewed above, there is inconsistent evidence for the recruitment of cognitive control during sentence processing, raising a question about the generalizability of cognitive control effects across different kinds of sentences. Our first premise (Pr1) explored this question by testing the same participants on two well-studied sentential ambiguities (SS and PA):

Pr1: Cognitive control will assist in the processing of sentences that contain conflict between interpretations, with possible differences between conflict types.

Based on available evidence and knowledge about neural streams, we predicted that conflict modulation effects would be detected for the SS conflict type because SS conflict sentences induce conflict between distinct (e.g. semantic and morphosyntactic) processing streams, and frontal cognitive control mechanisms could be useful for integrating across these streams. Evidence from different behavioural and neural paradigms (e.g. [16,27]) supports this prediction.

For the PA conflict type, our prediction was less certain. Previous studies have shown that cognitive control can influence eye movements and actions corresponding to PA sentences [15,17]. However, in these studies, participants acted out the instructions provided by ambiguous and unambiguous ‘Put’ sentences. Thus, processing PA sentences in those contexts imposed additional task demands on the participants—to plan actions, and to revise any incorrectly planned actions. Prior research suggests that embedding language processing in the context of a task can recruit additional brain networks and functions, including cognitive control, compared with when comprehension is task-free [31,32]. Therefore, it is unclear whether processing PA sentences in a context that requires no other action than comprehension would necessarily recruit cognitive control. We considered the possibility PA conflict sentences might primarily induce syntactic reanalysis, which could be handled by the syntactic processor without the engagement of cognitive control.

### 1.2. Individual differences

In addition to potentially differential engagement of cognitive control by different sentence structures, we also considered the possibility that individuals could differ in how much they use cognitive control for online sentence comprehension. If some individuals recruit cognitive control to resolve conflict during online processing and others do not, that could potentially explain why some studies have not found a convincing link between cognitive control and sentence comprehension when analysing specific individuals (e.g. [8]) or a group of participants as a whole (e.g. [7]). It is also consistent with evidence showing that activity within a widely studied cognitive control network (the multiple demand system) during language comprehension shows low inter-subject correlations (i.e. high variability across individuals) [5].

Consideration of such individual differences implicitly acknowledges the possibility that we might not find group-level conflict modulation effects as hypothesized under Pr1. Rather, some individuals might show modulation and others might not. We hypothesized that people with better cognitive control might be more able and/or efficient in allocating conflict resolution resources for resolving conflict during sentence processing, leading to Pr2:

Pr2: Cognitive control will differentially influence online processing based on individuals' cognitive control abilities, with possible differences between conflict types.

Individual differences in cognitive control could potentially impact sentence processing in two different ways. First, at the trait level, it could be the case that people who are better at cognitive control might be better at processing sentences containing conflict overall. Previous studies indicate that there are stable and detectable trait-level differences in cognitive control [33]. If these differences impact sentence processing, then there should be reading time differences between individuals during critical regions in conflict sentences. This is the typical approach taken by individual difference studies (e.g. [34]). Our new contribution here is that we examined this relationship for two different kinds of conflict (SS and PA) within the same participant population, which allowed us to determine if there is a relationship with trait-level cognitive control scores for both SS and PA conflict, or whether there is an effect for SS but not PA under conditions where there was no overt online processing task other than comprehension.

In addition to trait-level differences, any given person's cognitive state can also vary depending on the time of day or the situational context. In fact, it has been suggested that the conflict modulation paradigm is particularly useful for measuring state-level differences within an individual because it involves examining how a sentence is processed differently following one kind of cognitive control trial versus another [35]. Are some people better at turning cognitive control on and off? This question is relatively novel and much less studied. We investigated whether there is a trait-by-state interaction, i.e. whether people with better trait-level cognitive control are also better at flexibly modulating the recruitment of cognitive control for sentence processing. If this were true, we should observe an interaction between trait-level cognitive control scores and the previous Stroop trial type (which is intended to influence the temporary cognitive control state of the individual) for conflict (but not no-conflict) sentences. Here too, we asked if the effect holds for both conflict types, or for SS but not PA.

Any effects of the cognitive control scores could go in either direction because better cognitive control could enable individuals to better detect and attempt to handle the conflict (leading to slower reading times) or be more efficient in resolving the conflict once detected (leading to faster reading times). We could potentially even observe slowing down and speeding up at different points in the sentence during online processing. Therefore, we framed Pr2 to be non-directional for both questions.

### 1.3. Offline comprehension processes and task demands

Cognitive control is a broadly relevant ability that impacts many aspects of how people adjust their behaviour to the current context and goals. The domain-general recruitment of cognitive control in response to challenging tasks and increased task demands has been well documented. In fact, some researchers have argued that such task-related adjustment might be cognitive control's main or only contribution to language tasks (e.g. [5]). Others have shown that online and offline language comprehension can dissociate in persons with aphasia, suggesting a differentiation between online sentence processing resources and offline ‘post-interpretive’ processes [36,37]. In the present study, we asked participants to answer a comprehension question after reading the sentence. This allowed us to query the final interpretation arrived at by the participants, but also to test the premise that cognitive control will influence offline comprehension broadly, dictated by task demands (Pr3).

Pr3: Cognitive control will differentially influence offline comprehension accuracy based on individuals' cognitive control abilities, whenever the offline task requires choosing between competing options.

For SS sentences, answering the question correctly required evaluating thematic roles (who did an action versus was affected by the action). For conflict sentences in particular, this required going against prepotent semantic expectations, regardless of whether the correct answer is Yes or No (e.g. Sentence: ‘During the therapy session, the psychiatrist was analysed by the sympathetic counselor’. Question: ‘Did someone evaluate the psychiatrist?’ Answer: Yes. Sentence: ‘At the Oscars, the host was teased by the famous celebrity’. Question: ‘Did the host pick on someone?’ Answer: No). For no-conflict sentences, the correct answer conformed to semantic expectations. We hypothesized that individuals who are better at cognitive control would show higher accuracy especially for conflict sentences. For PA sentences, whether conflict or no-conflict, the comprehension question required participants to choose between three response options, two of which were potential destinations for ‘put’ actions mentioned in the sentence (e.g. Sentence: ‘In the kitchen, Cassandra said put the banana in the pantry into the bowl and grab a knife’. Question: ‘Where did the banana end up?’ Answer options: Other, Bowl, Pantry). We hypothesized that individuals who are better at cognitive control will be better at choosing between multiple options. Thus, unlike the uncertain predictions for online processing (see above), we expected that PA sentences will definitively show a correlation between cognitive control ability and higher offline accuracy.

### 1.4. Summary and additional clarifications

The central thesis guiding this study is that cognitive control may be variably recruited by different individuals for different situations, but that this variability will be systematic depending on the type of sentential ambiguity or conflict, individual differences in cognitive control, and task demands. This led to three premises (Pr1, Pr2, Pr3). Pr1 and Pr2 hypothesized that online conflict processing and modulation effects would be observed at the group level (Pr1), or show individual variation based on participants' cognitive control abilities (Pr2). Additionally, it is possible that any observed effects might differ between the two types of conflict (SS and PA). For offline processing, any task situation that entails a challenging decision between multiple competing response options should benefit from the recruitment of cognitive control, leading to better performance (Pr3). These effects should be observable for both conflict types.

Testing Pr1 could make a novel contribution to the literature on conflict modulation by allowing us to look for group-level modulation effects during online processing with two different kinds of conflict sentences within the same population using a reading comprehension without any action task (cf. act-out tasks in previous studies). Individual differences analyses for Pr2 can clarify if trait-level cognitive control differences between people influence sentence processing and/or interact with the state-level manipulation (previous Stroop trial type). Evaluation of a trait-by-state interaction, in particular, could be a novel contribution. Finally, individual differences analyses for offline measures under Pr3 offered a test of our proposal that the utility of cognitive control for language-related behaviours is versatile—while Pr1 and Pr2 allow for finding that cognitive control is more likely to be used for some kinds of conflict over others during online comprehension, Pr3 proposed to test cognitive control's broader utility for offline comprehension and decision-making across different conflict types. It is worth noting here that real-life language use involves ‘tasks’ too, for example, understanding people under noisy situations or choosing between alternative actions. Thus, while the distinction between comprehension with and without an explicit task is important theoretically, the adjustment of cognitive control according to task demands need not diminish its importance for language.

The domain-generality of cognitive control functions used for language has been widely discussed and disputed (e.g. [6,38–40]). Between the two extreme positions (all general or all specific), a middle ground, namely sub-specialization of general-purpose cognitive control for different representations within different frontal regions, has also been proposed (e.g. [40] and references therein). This study did not address the question of domain-generality. We used a Stroop task involving linguistic content to investigate conflict modulation, because this task has been used in multiple previous studies involving SS and PA sentences [15,16]. We return to the question of how future studies could extend the findings of the current study to explore domain-generality, under the Discussion section.

Before turning to the methods, we would like to highlight and clarify some procedural details. First, we have already normed the sentence stimuli to confirm that they lead to the expected conflict effects. We have also piloted the study, which allowed us to conduct an exploratory factor analysis and calculate the power for detecting critical effects. These are described under Norming and piloting. Second, table 1 describes the proposed analyses that were used to test each hypothesis. We analysed each word in the critical disambiguating region separately because there could be temporally transient conflict effects that are present at some words and not others. Appendix A shows the list of critical stimuli (normed and piloted). The bolded words indicate the words in the disambiguating region that were analysed. Because we tested three separate models at three different words, we have indicated which effects are significant uncorrected (*p* < 0.05) and with Bonferroni correction (*p* < 0.017). Third, we used separate models for SS and PA because the two kinds of sentences have unavoidable differences that preclude direct comparison between them in a combined model. These differences include where in the sentence conflict occurs, how far into the sentence conflict occurs, and the syntactic structures and meanings of the sentences. Given the use of separate models, any claims about differences between the two conflict types needs to be moderated. We have expanded on this in the Discussion. Finally, individual differences analyses of cognitive measures are subject to reliability concerns [41]. Therefore, we used multiple tasks that tap the same construct and conducted exploratory factor analysis to extract factor scores [38]. For the dependent variables (sentence reading times and accuracies), we have reported split-half reliabilities and split-half correlations between random slopes. The norming and pilot experiments indicated that this approach is viable and interpretable (see below).

**Table 1.** Study design.

question	stated hypothesis	alternative hypothesis	analysis plan	interpretation given different outcomes
Does cognitive control influence the online processing of sentences that contain conflict? Do these effects generalize across different types of conflict?	**Hypothesis 1**. Significant conflict modulation effect at words in the disambiguating region for SS. For PA, there may or may not be a significant conflict modulation effect.	No significant group-level conflict modulation effect for SS if there is large individual variability in how cognitive control is used during online processing.	Separate mixed-effects regression models for each conflict type (SS, PA) and critical word. DV = length-adjusted reading time. Fixed effects = previous Stroop trial type, current sentence trial type, interaction between the two, trial number. Random effects: intercepts and slopes for participants and items. Trial number^a^ is a continuous variable that was centred. Models with random slopes did not converge in the pilot as well as in the pre-registered experiment.	If the SS models yield a significant Stroop-by-sentence-type interaction, we will claim support for Hypothesis 1. If there is no significant interaction for the SS models, we will conclude that there is no evidence for the stated hypothesis. If there is an interaction for both the SS and PA models, we will conclude that the effect of cognitive control generalizes across these two types of conflict. If there is an interaction for SS but not PA, we will conclude that the effect of cognitive control can differ for different sentential ambiguities.
Does cognitive control differentially influence online processing based on individuals' cognitive control abilities? Do these effects generalize across different types of conflict?	**Hypothesis 2a.** Trait-by-state interaction for SS. Three-way interaction between individuals' cognitive control scores, previous Stroop type and the current sentence type, resulting from a two-way interaction for SS conflict but not SS no-conflict sentences. This would indicate differential correlations for incongruent sentences following congruent versus incongruent Stroop. For PA, we may or may not find such effects.	There will be no trait by-state interaction for SS if individual differences in cognitive control are not relevant for how cognitive control is modulated under different situations.	Separate mixed-effects regression models for each conflict type (SS, PA) and critical word. DV = length-adjusted reading time. Fixed effects = previous Stroop trial type, current sentence trial type, cognitive control, interaction between the three, working memory, trial number. Random effects: intercepts and slopes for participants and items. Cognitive control scores, working memory scores, and trial number are continuous variables that were centred. Models with random slopes did not converge in the pilot as well as in the pre-registered experiment. Note: working memory is included as a covariate to evaluate whether any effects of cognitive control are present after controlling for working memory. The effects of working memory are listed in the results tables, but we have not discussed them in the text because the present study does not address any claims regarding working memory *per se*.	If the SS model yields a trait-by-state interaction, we will claim support for Hypothesis 2a. If the SS model yields a trait effect but not a trait-by-state interaction, we will claim support for Hypothesis 2b. If both the SS and PA models yield an effect, we will conclude that the effect generalizes across SS and PA. If the SS but not the PA model yields an effect, we will conclude that the effect can differ for different sentential ambiguities. All other patterns of results will lead to no support for Hypothesis 2.
	**Hypothesis 2b.** Trait effect but no trait-by-state interaction for SS. Two-way interaction between individuals' cognitive control scores and current sentence type, resulting from a correlation between reading times and individuals' cognitive control scores for all incongruent but not congruent sentences, whether preceded by congruent or incongruent Stroop. For PA, we may or may not find such effects. Note: a correlation with cognitive control in either direction is compatible with the hypotheses because better cognitive control could potentially make individuals slow down at critical points during online processing and speed up at others.	There will be no trait effect for SS if individual differences in cognitive control are not relevant for how incongruent sentences are processed.		
Does cognitive control help broadly with offline performance whenever the task involves choosing between competing options?	**Hypothesis 3.** Negative correlation between cognitive control scores (errors) and offline comprehension accuracy for both SS and PA. Individuals with better cognitive control should show higher accuracy.	Cognitive control will not correlate with offline comprehension accuracy if it is not relevant to offline comprehension.	Separate multiple logistic regression models for each conflict type (SS, PA). DV = offline comprehension accuracy (0 or 1) for congruent and incongruent sentences. IVs = current sentence trial type, cognitive control, interaction between the two, working memory, trial number. Cognitive control scores, working memory scores, and trial number are continuous variables that were centred. Note: mixed-effects models with random effects did not converge in the pilot as well as in the pre-registered experiment.	If regression models for SS and PA yield a significant negative correlation with cognitive control factor scores, or an interaction between cognitive control and current sentence type, we will claim support for Hypothesis 3. If neither model shows a significant negative correlation or interaction with current sentence type, we will conclude that there is no evidence for the stated hypothesis. Note: we do not expect positive correlations (more cognitive control errors associated with higher offline comprehension accuracy), because that is highly unlikely both theoretically and empirically.

^a^We included trial number in all models to account for variation in performance as the experiment progresses (e.g. participants could get faster due to more practice with the task, or slower due to fatigue).

## 2. Methods

### 2.1. Participants

A total of 104 (49 female, 54 male, 1 non-binary)^2^ right-handed native English speakers ages 18–35 and located in the United States took part in this study. They were recruited under a protocol approved by the institutional review board at The George Washington University. Participants were recruited using Prolific, an online research platform developed specifically for research. The platform uses transparent and ethical recruitment procedures, and includes features for screening participants [42]. Demographic information was collected independent of the research study, minimizing concerns about misrepresentation by the participants. For the present study, the inclusion criteria were: English monolingual, right-handed, normal or corrected to normal vision, undergraduate degree or higher education, and no language-related disorders, head injury, cognitive or mental conditions. An English monolingual participant was defined as a person who is fluent in English only, based on the following questions: ‘Are you an English-speaking monolingual, that is, are you fluent only in English? Were you raised monolingual? Or are you also fluent in any other language(s)?’

Multi-day experiments are subject to dropout, whether conducted in the laboratory or online. To help control attrition, we used Prolific's messaging features to send participants multiple text reminders about completing the second day of testing. We paid the participants after they completed both days. The dropout rate was 19% in the pilot experiment and 24% in the pre-registered experiment. Given the large pool of participants available, we were able to replace the participants who dropped out and complete pilot and pre-registered testing in a reasonable amount of time.

We collected accuracy on all tasks and could therefore verify whether participants were attending to the tasks. In the pilot as well as the pre-registered experiment, mean accuracy on the cognitive control tasks was greater than 90%. We excluded participants who did not score above chance on the comprehension questions following the experimental sentences in order to ensure that only data from those who were attending to sentence reading were included (3.8% excluded in the pilot and less than 1% excluded in the pre-registered experiment). Two of the working memory paradigms involved dual tasks. The established protocol requires exclusion if participants do not meet the minimum accuracy of 85% for the secondary task [43]. These tasks and inclusion criteria are challenging independent of testing location and are expected to result in the exclusion of 15% or more of the participants [43]. Therefore, as anticipated from the pilot, we had some exclusions. Specifically, 41 participants were excluded because their comprehension accuracy on the sentence trials was below chance (less than 66/112) or they did not meet the criteria on the reading and operation span tasks. Together, these accuracy checks helped ensure data quality.

Given that this study was conducted online, we were able to attest to participants' performance but not to how exactly they did the cognitive tasks. That said, experimenters can seldom ensure that participants are completing tasks as instructed and only as instructed. Adult participants may choose to use different strategies (e.g. chunking) that are not visible to others. Two features of this study address this potential concern. First, we used factor scores extracted from factor analyses of multiple tasks that tap the same construct. Variable strategy used by the participants across tasks could introduce noise and thereby increase the risk of a type II error, but it is unlikely to lead to a type I error. Second, we were able to compare the pre-registered experiment results with the pilot results and thereby evaluate whether the findings are replicable using different sets of participants in the two experiments.

### 2.2. Day 1 tasks

To test whether cognitive control influences online sentence comprehension, we pseudorandomly interleaved the colour-word Stroop task with a self-paced reading task involving two different kinds of conflict (SS versus PA). We also included subject and object relative clause sentences that served as fillers. All stimuli were presented using Psychopy v. 2021.1.2 [44] and Pavlovia.org [45]. In the Stroop task, participants saw colour words on the screen (blue, green, yellow, brown, orange, red) in different font colours (blue, green, yellow). They were asked to press a button on the keyboard that corresponds with the font colour (J for blue, K for green and L for yellow). Word meaning and font colour matched in the congruent condition (e.g. BLUE in blue font) and did not match in the incongruent condition (e.g. ORANGE in blue font). To avoid conflict at the response level, we ensured that the word stimulus in the incongruent condition (e.g. ORANGE) was not a possible response. Therefore, akin to the conflict in the sentence reading task, the Stroop task had conflict at the representational level only [15,16,46]. Each trial began with a central fixation cross (500 ms), and then a colour word with the response options printed at the bottom of the screen was displayed (1000 ms or until the participant responds).

In the self-paced reading task, participants read sentences word by word and answered comprehension questions. As described earlier, there were two types of conflict-inducing sentences: syntax semantics (SS), and phrase attachment (PA). Half of the SS sentences were congruent (e.g. *For the official portrait, the queen was painted by the talented artist*) and half incongruent (e.g. *In the studio, the artist was painted by the talented students*). Incongruent SS sentences required resolving conflict between syntax and semantics, and favouring the interpretation given by syntax over the one suggested by semantic plausibility. For the PA sentences, a third were categorized as congruent (e.g. *In the mansion, Autumn said put the rug that's on the hardwood floor into the cupboard and start vacuuming*), a third as incongruent^3^ (e.g. *In the mansion, Autumn said put the rug on the hardwood floor into the cupboard and start vacuuming*), and a third as filler (e.g. *Working at the take-out restaurant, Stephanie said put the rice in the wok before you add the sauce*). Incongruent PA sentences were expected to lead to temporary conflict in interpreting the first prepositional phrase after ‘put’, which might initially be interpreted as the destination and must later be reinterpreted as a modifier. In congruent PA sentences, the presence of ‘that's’ unambiguously indicated the modifier interpretation, thereby leading to no conflict. In filler PA sentences, the first prepositional phrase after put (e.g. *in the wok*) was the correct destination. This was done to ensure that not all PA sentences resolve toward a modifier interpretation. We also included relative clause (RC) sentences, half subject relative and half object relative. The main clause of these sentences used the same set of verbs as the SS sentences but in the active voice, thereby serving as fillers that counterbalance the use of the passive voice in all SS sentences. The full set of stimuli is listed in appendix A.

In each sentence trial, the sentence was displayed in two consecutive segments. Participants first saw the initial contextual phrase as a whole (e.g. *For the official portrait*), and then the main part of the sentence (e.g. *the queen was painted by the talented artist*) word by word. During the word-by-word segment, participants initially saw dashes in place of the words. As they pressed the spacebar to progress through the sentence, each word appeared at the appropriate location. The number of dashes at a given location equalled the character length of the corresponding word. Only one word was visible on the screen at any given time. When participants moved forward, the current word was displayed and the previous word reverted to dashes.

All sentences were left aligned, displayed at the centre of the screen, and presented in a single line. A comprehension question appeared on the screen in its entirety once the participant moved past the final word of a sentence. Each trial began with a central fixation cross (500 ms), followed by the contextual phrase (until the participant responds), the main part of the sentence word by word, and finally the comprehension question. Participants pressed yes or no by pressing the J or K key for the comprehension question after SS and RC sentences. Comprehension questions for PA sentences had three answer options (J, K and L). These comprehension questions also served as an attention check throughout the experiment. If a participant fell below chance performance, we excluded them from the second day of testing.

Stroop and sentence trials were pseudorandomly interleaved such that there were 84 Stroop-to-sentence pairs (28 Stroop-to-SS, 28 Stroop-to-PA, plus 28 Stroop-to-RC). For SS and PA, there were seven pairs of each of the following conditions: congruent Stroop followed by congruent sentence (CC), congruent Stroop followed by incongruent sentence (CI), incongruent Stroop followed by congruent sentence (IC) and incongruent Stroop followed by incongruent sentence (II). Congruent and incongruent Stroop trials were followed equally often by congruent and incongruent sentences of different types. Thus, participants were not able to predict the condition or type of the sentence based on the Stroop trials. To further prevent participants from predicting what would come next, we added 44 filler Stroop trials and 28 filler sentence trials. Overall, the interleaved structure comprised all kinds of transitions: Stroop-to-sentence, Stroop-to-Stroop, sentence-to-Stroop, and sentence-to-sentence. Each participant completed 84 Stroop and 84 sentence trials that were part of a pair (as described above), 44 filler Stroop trials (half congruent and half incongruent) and 28 filler sentence trials that preceded and followed other Stroop or sentence trials.

Four lists were used to counterbalance the assignment of sentences to conditions. Any given congruent or incongruent sentence was preceded by a congruent Stroop trial in two lists and by an incongruent Stroop trial in the other two lists. An additional four lists were created by reversing the order of trials in the first four lists. Each participant was randomly assigned to a list and no participant read both the congruent and incongruent versions of the same sentence. Each list was separated into four blocks with breaks in between. Each block consisted of 60 trials. Order was pseudorandomized such that each conflict modulation condition (CC, CI, IC, II) appeared a maximum of two times in a row and each conflict type (SS, PA, relative clause) also appeared no more than two times in a row. Each block contained at least one trial per conflict modulation condition for each conflict type. The correct response for answering the comprehension question was balanced within each conflict type.

Before starting the interleaved portion of the study, each participant completed 5 practice Stroop trials, which they repeated until they reached 80% accuracy. Subsequently, they completed seven practice sentence trials. Participants had to score 80% or above on the comprehension questions following the sentences in order to move on from the practice. Following the Stroop-only practice and sentence-only practice, participants completed 12 interleaved Stroop and sentence trials (five Stroop and seven sentence). Again, participants had to score 80% or above to move on. Practice was repeated if that was not the case. Sentences seen during practice were not used in the main experiment.

### 2.3. Day 2 tasks

During the second session, participants completed three working memory (reading span, operation span, backwards digit span (BDS)) and three cognitive control (Stroop, AX-CPT^4^ and Flanker) tasks in the following order: Stroop, reading span, Flanker, backwards digit span, operation span and AX-CPT. All tasks were administered using PsychoPy on the Pavlovia platform. The second session happened between 1 and 7 days from the first session. Although the Stroop task was used in both sessions, this occurred on different days and in different contexts, and contained different combinations of trials. On Day 1, the Stroop task was interleaved with sentence reading and contained 64 congruent and 64 incongruent trials. On Day 2, the Stroop task was a standalone task that contained 12 congruent, 60 incongruent and 72 neutral trials (see below). The Day 2 Stroop task was also entered into a factor analysis containing other cognitive control tasks. Therefore, we did not expect practice effects to substantively impact the individual differences analyses.

#### 2.3.1. Stroop

Participants saw words on the screen (blue, green, yellow, brown, orange, red) in different font colours (blue, green, yellow). They were asked to press a button on the keyboard that corresponded with the font colour (J for blue, K for green and L for yellow). Word meaning and font colour matched in the congruent condition (e.g. BLUE in blue font) and did not match in the incongruent condition (e.g. ORANGE in blue font). This required participants to inhibit selecting what the text said and select what the font colour was instead. The neutral condition used a line of asterisks instead of a colour word (e.g. ***** in blue font). On each trial, participants saw a fixation cross (500 ms) followed by the stimulus (1000 ms). They were asked to use their right hand to respond as quickly and as accurately as possible. Participants received practice with 8 trials (three congruent, three incongruent and two neutral) along with feedback. The practice block was repeated until participants reached 80% accuracy. The critical block consisted of 144 trials (12 congruent, 60 incongruent and 72 neutral). No feedback was given during these trials. We collected response time (RT) and accuracy on each trial.

#### 2.3.2. Reading span

In the reading span task [43], participants read sentences and rated their plausibility (e.g. The woman took her banana for a ride. Does this make sense?). Half of the sentences were plausible and half were implausible. After each sentence, they saw a letter (F, H, J, K, L, N, P, Q, R, S, T or Y) for 800 ms. They were asked to recall these letters in the correct order after completing each set of sentences for a given span length (span length 4 will have four pairs of sentences and letters, 6 will have six pairs, and so on). Participants used a mouse or touchpad to select letters in the correct order from a 4 × 3 matrix. They could take as long as they wanted and received feedback on how many letters were correctly recalled.

Prior to doing the task, participants received practice. The practice was broken down into three parts. First, they received four practice trials for the letter recall task (two trials of two letters and two trials of three letters) with feedback on the accuracy of each trial. Then they received 15 practice trials for the sentence plausibility judgement task (seven plausible and eight implausible). They were asked to use the mouse to select whether a sentence was plausible or implausible as quickly and as accurately as possible, and then they received feedback on the accuracy of each trial. Reading time was recorded for the purpose of computing the mean reading time for each participant. The practice sentence trials were repeated if accuracy was below 85%. The final portion of the practice combined the sentence plausibility judgement and letter recall tasks and imposed a time limit for the sentence presentation. Sentences were displayed for the mean reading time plus 2.5 s.d. computed from the final sentence practice passed. If a participant took longer than the maximum allowed time, the plausibility response window was skipped and the trial was marked as incorrect. Letters were displayed for 800 ms for all participants. Participants received three practice trials at span length 2 (i.e. three sets of two sentence-letter pairs). Testing moved on to the critical trials regardless of performance on this portion of the practice.

After practice, the critical trials were presented in a fixed random order of span length such that all participants received the same order (5, 4, 3, 6, 7, 5, 3, 4, 7, 6, 7, 5, 4, 3, 6). Across all trials, there were two trials at each span length. As during the final portion of practice, sentences were presented for the duration based on each participant's mean and standard deviation of reaction times. Participants were told to keep their accuracy for the sentence judgement portion of the task above 85% during the critical trials. Accuracy was displayed in the top right of the screen after the recall portion of the task. Participants were also given feedback on their accuracy of recall.

For the analyses, we computed a partial span score for each participant. The partial span score was calculated by dividing the number of correctly recalled items in a trial by the total number of items in a trial (e.g. 3/5 = 0.6). After each trial received a partial span score, the mean partial span score was calculated as the sum of all trials’ partial span scores divided by the total number of trials (e.g. 12.4/15 = 0.83).

#### 2.3.3. Flanker

In the Flanker task, participants saw rows of five arrows on the screen and indicated the direction of the middle arrow by pressing a button (J for left, K for right). There were two conditions. In the congruent condition, the middle and surrounding arrows pointed in the same direction (e.g. >>>>>). In the incongruent condition, they did not match (e.g. >><>>), thereby requiring cognitive control to selectively attend to the middle arrow and ignore interference from the surrounding arrows. Practice consisted of 12 trials (four congruent, eight incongruent) with feedback. Practice was repeated if participants scored less than 80% correct. After practice, participants completed 80 critical trials (40 congruent, 40 incongruent). No feedback was given during the critical portion of the task. We collected RT and accuracy on each trial.

#### 2.3.4. Backwards digit span

In this task, participants saw single-digit numbers displayed one by one on the screen and then were asked to recall the digits in the reverse order that they were presented in. Participants typed out their responses using a keyboard. For practice, participants received three trials with a span length of 2. If a participant got at least two out of the three practice trials correct, they moved on to the critical trials. Otherwise, they repeated the practice until they met this criterion. Critical trials started at span length 3 and could go up to a maximum span length of 12. There were three trials at each span length. If a participant was correct on at least two out of three trials at a given length, testing moved on to the next span length. Conversely, testing stopped whenever participants got less than two out of three correct at a given span length. The BDS score for any given participant was the last length at which they obtained at least two correct trials (e.g. ⅔ correct on span 6 and ⅓ correct on span 7 would mean a span score of 6).

#### 2.3.5. Operation span

The operation span [47] task was similar to the reading span task except for swapping the sentence judgement task with an arithmetic operations task. Participants were presented with an arithmetic equation (e.g. (2 × 5) + 10 = ?) and then a number. They judged whether that number (e.g. 15) was the right solution for the equation. After each operation trial, they saw a letter (F, H, J, K, L, N, P, Q, R, S, T or Y) for 800 ms. As with the reading span task, they were asked to recall sets of letters of increasing span length. Critical trials were presented in a fixed random order (4, 6, 3, 7, 5, 3, 5, 6, 4, 7, 5, 7, 4, 6, 3) for each participant. The procedure (including practice) and scoring were identical to the reading span task.

#### 2.3.6. AX-CPT

In this task, participants responded to letter pairs presented sequentially on the computer screen. They were asked to press the J button when they saw the letter pair AX and the K button for all other pairs. The task contained four different conditions (AX, BX, AY and BY). The AX condition was the most frequent (70% of the trials = 105). The other 30% were split evenly across the BX, AY, and BY conditions (15 trials each). In the BX and BY conditions, the first letter could be any letter except A, I, L or X. Since AX (congruent) was the most frequent condition, the nature of the task meant that in the AY condition (incongruent), how accurately and efficiently participants could withhold pressing J (and respond with a K instead) indexed cognitive control.

Participants received eight practice trials with feedback after each trial. They moved on from practice after they got at least 80% correct. The probe and target letters were presented for 250 ms with a blank screen separating them for 500 ms. There was no time limit for the response, but participants were instructed to respond as quickly and accurately as possible. After each pair of letters, the screen displayed ‘******* end of this trial *******’ for 500 ms to indicate that the previous trial was over. After practice, participants completed 150 critical trials, with no feedback. We recorded accuracy and RT on each trial.

### 2.4. Dependent measures and analyses

Pr1 is about online sentence processing. The dependent measure for this hypothesis was length-adjusted reading time for different words in the disambiguating region of the sentence, which include the disambiguating word (Word0) plus the next two words (Word1 and Word2). These are bolded in the list shown in appendix A. We used mixed-effects models to examine the effect of previous Stroop trial type, current sentence trial type, and the interaction between the two variables on reading times. Pr2 is about the effect of individual differences in cognitive control on how people use cognitive control during online sentence processing. The dependent measure was length-adjusted reading time for different words in the disambiguating region of the sentence. Mixed-effects models tested whether trait-level cognitive control differences correlate with reading times and whether this interacts with previous Stroop trial type (a trait-by-state interaction). For Pr1 and Pr2, trials where participants answered the previous Stroop trial incorrectly could have different consequences on the online processing of the subsequent sentence (e.g. post-error slowing) and complicate interpretation. Therefore, we have reported results from all eligible trials independent of previous Stroop accuracy, and from the subset of trials that follow correct Stroop trials only. Finally, Pr3 is about the effect of individual differences in cognitive control on offline comprehension accuracy. We tested if cognitive control affects offline accuracy broadly by helping participants choose between competing options. Please see table 1 for a summary of the stated and alternative hypotheses.

### 2.5. Norming and piloting

#### 2.5.1. Norming results

We selected SS and PA stimuli using two separate web-based self-paced reading studies. For SS sentences, 80 participants read 240 sentences word by word. This included 60 passive sentences containing syntax-semantics conflict, 60 passives with no conflict and 120 active fillers. Sixty verbs appeared four times, once each in conflict and no-conflict passives and twice in active fillers. From the 60 verbs, we picked 28 verbs that showed faster reading in the disambiguating region (i.e. a negative estimate in the mixed model analysis) for no-conflict compared with conflict sentences. For these 28 verbs, the effect of conflict was significant at words 0, 1 and 2 (i.e. the verb and the two words following the verb. *t*-values < −4, *p*s < 0.001). For the PA sentences, 80 participants read 60 sentences word by word. This included 15 ‘put’ sentences with conflict, 15 with no conflict and 30 fillers where the prepositional phrase resolved to a destination (instead of a modifier). All critical sentences used the verb ‘put’. There were 60 different scenarios counterbalanced across participants from which we chose 28 scenarios that showed the largest difference between no-conflict and conflict sentences in the disambiguating region (similar to SS sentences). For these 28 scenarios, the effect of conflict was not significant at word 0 (preposition; *p* > 0.6), marginally significant at word 1 (determiner; *p* = 0.09) and significant at word 2 (noun; *t* = −5.2, *p* < 0.001). The final items are shown in appendix A. These items were used in the pilot experiment and in the pre-registered experiment.

#### 2.5.2. Pilot results

We collected pilot data using the procedure described in Methods. Below, we describe the results for each hypothesis stated in table 1. The formulae for all analyses can be found in appendix B.

##### 2.5.2.1. Hypothesis 1: significant conflict modulation effect in the disambiguating region for SS and possibly PA

For the first hypothesis, we analysed reading times in the disambiguating region for each conflict type (SS and PA) separately. Eighty adults participated. Three were excluded from all the analyses (Hypotheses 1, 2 and 3) because their comprehension accuracy on the sentence trials was below chance (less than 66 correct out of 112 comprehension questions). Data from the remaining participants (*N* = 77; 40 female) were analysed using the following procedure. First, raw RTs that were below 100 ms and above 2000 ms were removed [48]. Second, we log-transformed the RTs^5^ and removed outliers using the mean absolute deviation (MAD) method (upper range: +3 s.d. from median, lower range: −3 s.d. from median; [50]). Together, this resulted in the exclusion of less than 1% of the trials. Finally, we length-adjusted the reading times [51]. For each participant, log-transformed reading times for the filler trials were regressed onto word length using linear regression. The slope and intercept from the model were used to residualize the reading times for the critical trials. These residualized or length-adjusted reading times served as the dependent measure in all the reading times analyses reported below.

For online processing of the SS sentences, our analysis focused on the length-adjusted reading times for the disambiguating verb (Word0) plus the following two words (Word1 and Word2) (e.g. bolded words in *‘During tax season the IRS employee was **audited by the** determined accountant*’). We constructed three separate mixed effects regression models (‘lmer’ function from the lmerTest package, v. 3.1–3 [52], in R, v. 3.6.0 [53]) for the three words (Word0, Word1 and Word2) with fixed effects of previous Stroop trial type, current sentence trial type, their interaction, trial number, and random intercepts for participants and items (see appendix B for the complete equations; we could not fit models containing random slopes due to convergence error). The effect of interest was the interaction between the previous Stroop trial type and the current sentence trial type (see appendix C for full model output). The analyses were carried out for all trials (i.e. irrespective of accuracy on the previous Stroop trial) and for trials where participants responded accurately on the previous Stroop trial (i.e. Stroop correct only). Results revealed no significant interactions for all three models (all trials—Word0: estimate = 0.01, s.e. = 0.03, *p* = 0.66; Word1: estimate = 0.05, s.e. = 0.03, *p* = 0.17; Word2: estimate = 0.02, s.e. = 0.03, *p* = 0.53; Stroop correct only—Word0: estimate = 0.02, s.e. = 0.03, *p* = 0.61; Word1: estimate = 0.05, s.e. = 0.03, *p* = 0.17; Word2: estimate = 0.02, s.e. = 0.03, *p* = 0.51).

The analysis of PA sentences (see appendix D for full model output) focused on the length-adjusted reading times for the disambiguating preposition (Word0) plus the following two words (Word1 and Word2) (e.g. bolded words in *‘Working at the valet stand Colin said put the car in the entrance **into the parking** lot and drive slowly’*). We did not find a significant interaction between the previous Stroop trial type and the current sentence trial for any of the models (all trials—Word0: estimate = 0.02, s.e. = 0.03, *p* = 0.54; Word1: estimate = −0.02, s.e. = 0.03, *p* = 0.59; Word2: estimate = 0.002, s.e. = 0.03, *p* = 0.96; Stroop correct only—Word0: estimate = 0.02, s.e. = 0.03, *p* = 0.58; Word1: estimate = −0.02, s.e. = 0.03, *p* = 0.55; Word2: estimate = 0.001, s.e. = 0.03, *p* = 0.99).

Thus, Hypothesis 1 was not supported by the pilot data. We calculated Bayes Factors based on the Bayesian information criterion (BIC) for all models to examine whether the pilot data are more likely under Hypothesis 1 (H1) than the null hypothesis (H0) (see appendix B for the complete equations; ‘lme4:lmer’ function from the lme4 package v. 1.1.25 [54]).^6^ We found strong to very strong evidence in favour of H0 compared with H1 for both SS (Word0: 41.2 times more in favour of H0 compared with H1, Word1: 17.1 times, Word2: 36.1 times) and PA (Word0: 37.9 times more in favour of H0 compared with H1, Word1: 39.6 times, Word2: 46 times) conflict types. However, we still tested Hypothesis 1 in the pre-registered study as planned (and have reported Bayes factors again).

##### 2.5.2.2. Hypothesis 2: significant effect of individual differences in cognitive control on online processing for incongruent SS and potentially incongruent PA sentences

For the Day 2 tasks (see Methods), we computed the following variables for each participant: mean accuracy and mean RT for the congruent and incongruent Stroop trials (Stroop), mean accuracy and mean RT for the AX and AY trials (AX-CPT), mean accuracy and mean RT for the congruent and incongruent Flanker trials (Flanker), backward digit span scores (BDS), partial reading span scores and partial operation span scores. Twenty-two participants were excluded because they scored less than 85% correct on the sentence and arithmetic probes in the reading and operation span tasks, as per standard practice for those tasks [43]. Therefore, all subsequent analyses (both Hypothesis 2 and Hypothesis 3) contained 55 participants (33 female).

**Figure 1.**
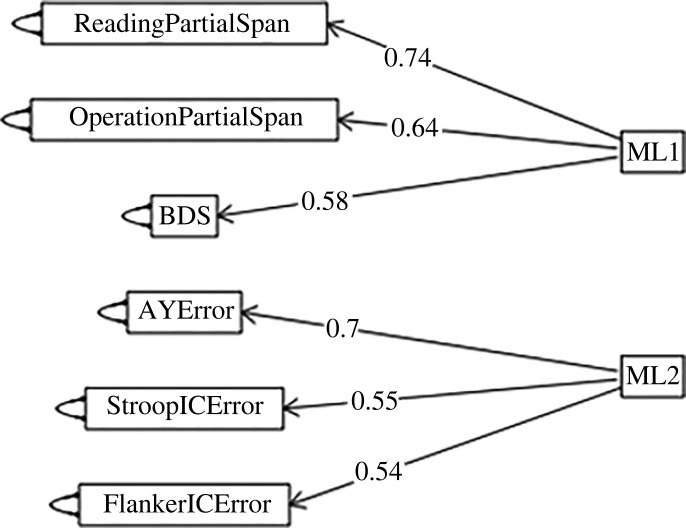
Path diagram from exploratory factor analysis (maximum likelihood with a varimax rotation). ML1 = working memory, ML2 = cognitive control.

The above-mentioned variables were subjected to exploratory factor analysis (EFA) using maximum-likelihood factoring and orthogonal varimax rotation (‘fafit’ function from the ‘psych’ package (v. 2.1.3; [55]) in R). The RT measures for the cognitive control tasks and the accuracy measures from the congruent trials of those tasks (errors from AX, Stroop congruent and Flanker congruent trials) did not load reliably on informative across-task factors. They were dropped. The remaining variables in the final factor analysis were accuracy measures from the incongruent trials of the cognitive control tasks (errors from the AY, Stroop incongruent and Flanker incongruent trials) and span scores from the working memory tasks. Kaiser–Meyer–Olkin (KMO) values for all of these variables were above 0.5 and the KMO measure was 0.7, indicating that the data were sufficient for EFA. Bartlett's test of sphericity revealed that there were patterned relationships between variables (*p* < 0.001). Using an eigenvalue cut-off of 1.0 and by looking at the scree plot, we retained two factors. The path diagram (figure 1) shows the factor loadings after rotation using a significant factor criterion of 0.4 (see appendix B for the complete equations). Reading partial span, operation partial span and BDS span loaded onto one factor (hereafter, working memory factor) and errors from the AY trials, Stroop incongruent trials, and Flanker incongruent trials loaded into another factor (hereafter, cognitive control factor).

We ran three separate mixed effects regression models (‘lmer’ function from the lmerTest package, v. 3.1–3 [52]) for the three words (Word0, Word1 and Word2) with fixed effects of previous Stroop trial type, current sentence trial type, cognitive control, their interaction, working memory, trial number and random intercepts for participants and items (we could not fit models containing random slopes due to convergence error; see appendix B for the model equations). The effects of interest were correlation with trait-level cognitive control and its interaction with the previous Stroop trial type (state), for incongruent but not congruent sentences. Similar to Hypothesis 1, we report the results from all trials and for trials following correct Stroop trials only. See appendix E for a table of the model results. We observed a significant three-way, previous Stroop by current sentence by cognitive control interaction at Word2 (all trials—estimate = 0.06, s.e. = 0.03, *p* = 0.03; Stroop correct only—estimate = 0.06, s.e. = 0.03, *p* = 0.02) but not at Word0 (all trials—estimate = 0.02, s.e. = 0.02, *p* = 0.30; Stroop correct only—estimate = 0.03, s.e. = 0.02, *p* = 0.26) or at Word1 (all trials—estimate = −0.02, s.e. = 0.03, *p* = 0.36; Stroop correct only—estimate = −0.02, s.e. = 0.03, *p* = 0.39). We further explored the significant three-way interaction at Word2 by analysing congruent and incongruent sentences separately. For the congruent sentences, there was no significant trait-level correlation (all trials—estimate = −0.03, s.e. = 0.02, *p* = 0.18; Stroop correct only—estimate = −0.03, s.e. = 0.02, *p* = 0.19) and no trait-by-state interaction (all trials—estimate = −0.02, s.e. = 0.02, *p* = 0.39; Stroop correct only—estimate = −0.02, s.e. = 0.02, *p* = 0.31). However, for incongruent sentences, there was a significant trait-level correlation (all trials—estimate = −0.05, s.e. = 0.02, *p* = 0.01; Stroop correct only—estimate = −0.05, s.e. = 0.02, *p* = 0.01) as well as a trait-by-state interaction (all trials—estimate = 0.04, s.e. = 0.02, *p* = 0.02; Stroop correct only—estimate = 0.04, s.e. = 0.02, *p* = 0.02). The trait-by-state interaction was the result of a significant correlation with trait-level cognitive control when incongruent sentences followed a congruent Stroop (all trials—estimate = −0.05, s.e. = 0.02, *p* = 0.008; Stroop correct only—estimate = −0.05, s.e. = 0.02, *p* = 0.008) but not an incongruent Stroop (all trials—*p* = 0.61; Stroop correct only—*p* = 0.61) trial.

Overall, the results were significant for incongruent but not for congruent sentences, suggesting that trait-level and trait-by-state effects were specific to cases of conflict. The trait-level correlation for incongruent sentences at Word2 suggests that individuals with better cognitive control (lower cognitive control error-based factor scores) spent more time reading the words in the disambiguating region, indicating that they detected conflict. The trait-by-state interaction for incongruent sentences at Word2 suggests that individual differences in trait-level cognitive control affected how individuals responded to state manipulations. When the previous Stroop trial was congruent, no pre-recruitment of cognitive control was expected for any individual. In this case, we observed slowed-down reading for people with better cognitive control at Word2. However, when the previous Stroop trial was incongruent and was expected to trigger the recruitment of cognitive control, individuals with better cognitive control were no longer slower at Word2. This suggests that they had resolved the conflict soon after disambiguation. In sum, the results suggest that individuals with better trait-level cognitive control were better at detecting conflict, and that triggering a state where cognitive control was engaged by the previous trial helped these individuals to resolve the detected conflict.

The corresponding analyses for the PA conflict type (see appendix F for the complete model results) revealed no significant trait-level correlation or trait-by-state interaction at any word (all *p*s > 0.1).

These pilot data support Hypothesis 2b by finding a significant correlation with cognitive control, and Hypothesis 2a by finding a significant interaction between individuals' cognitive control scores and the previous Stroop trial type at Word2 for incongruent SS sentences. Individual differences in cognitive control correlated with differential processing of incongruent SS sentences (a trait effect) and this effect was modulated by the cognitive control state of the participant as manipulated by the previous Stroop trial (a trait-by-state interaction). Please note that these trait and trait-by-state interactions were not significant with Bonferroni correction. Therefore, it was important to see if these effects replicated in the pre-registered experiment with a new sample of participants (see Results and Discussion). Neither a trait nor a trait-by-state interaction was observed for PA sentences, suggesting that these effects may not generalize to all conflict types.

##### 2.5.2.3. Hypothesis 3: significant effect of individual differences in cognitive control on offline comprehension for both SS and PA conflict types

Offline comprehension accuracy was modelled with logistic regression (glm function in R v. 3.6.0). Analysis of SS sentences (see appendix G for the complete results) revealed a significant correlation with cognitive control (estimate = −0.27, s.e. = 0.13, *p* = 0.04). For PA sentences (see appendix H for the complete results), the parallel analysis also found a significant correlation (estimate = −0.73, s.e. = 0.13, *p* < 0.001). In both cases, individuals with better cognitive control showed higher comprehension accuracy for all sentences, congruent and incongruent. There were no significant interactions between cognitive control and current sentence trial type (congruent versus incongruent; *p*s > 0.3).

These data support Hypothesis 3 because they demonstrate a broad effect of individual differences in cognitive control on choosing between competing options in the offline tasks. Unlike the online processing effects for Hypothesis 2, these effects were reliable for PA as well as SS, which is consistent with cognitive control being important whenever there is a challenging offline decision (choosing between three possible answers for PA and two possible answers for SS).

### 2.6. Reliability analysis

We estimated the internal consistency of the dependent variables (raw reading times for Hypotheses 1 and 2, accuracy for Hypothesis 3) using a permutation-based split half approach (‘splithalf’ function from the splithalf package, v. 0.7.1 [56]) with 5000 random splits. For Hypothesis 1 (*N* = 77), the Spearman–Brown (SB) corrected split-half reliability scores for SS sentences were *r*_SB_ = 0.70, 95% CI [0.59,0.79] at Word0, *r*_SB_ = 0.80, 95% CI [0.70,0.88] at Word1, and *r*_SB_ = 0.73, 95% CI [0.63,0.82] at Word2, and for PA sentences were *r*_SB_ = 0.78, 95% CI [0.70,0.85] at Word0, *r*_SB_ = 0.83, 95% CI [0.76,0.89] at Word1, and *r*_SB_ = 0.68, 95% CI [0.56,0.78] at Word2. For Hypothesis 2 (*N* = 55), split-half reliability scores for SS sentences were *r*_SB_ = 0.73, 95% CI [0.59,0.83] at Word0, *r*_SB_ = 0.72, 95% CI [0.59,0.82] at Word1, and *r*_SB_ = 0.74, 95% CI [0.61,0.83] at Word2, and for PA sentences were *r*_SB_ = 0.77, 95% CI [0.67,0.85] at Word0, *r*_SB_ = 0.84, 95% CI [0.75,0.90] at Word1, and *r*_SB_ = 0.64, 95% CI [0.48,0.77] at Word2. For Hypothesis 3 (*N* = 55), the split-half reliability score for SS sentences was *r*_SB_ = 0.65, 95% CI [0.47,0.78] and for PA sentences was *r*_SB_ = 0.76, 95% CI [0.62,0.85]. In summary, the dependent variables showed acceptable to excellent levels of reliability (0.64–0.84).

The above analysis suggests that reading times measured in our web-based self-paced reading paradigm can reliably distinguish between individuals who read faster versus slower. To evaluate whether differences in reading times between conflict and no-conflict sentences are also reliable, we chose to evaluate whether the random slopes for subjects were internally consistent (see [34]). This approach derives estimates from models that take into account the multiple factors affecting reading times within a paradigm, including trial number and other condition manipulations. We used models of the norming data because the norming experiment was akin to typical sentence processing studies in that each participant read conflict and no-conflict sentences and we measured reading time differences between the two conditions. The pilot data are not ideal for this purpose because the sentences were embedded within the context of Stroop trials, which was hypothesized to modulate reading times differently for different individuals. Additionally, mixed models containing random slopes did not converge for the pilot data. For the items selected for the experiment from the norming study, we split the data into 100 different permutations of halves (initial_split function, rsample package, v. 0.1.1). For each half, we extracted the random subject slopes for the conflict manipulation from the mixed model using the ranef function and then computed the Pearson correlation between the random slopes from the two halves [34]. The average correlation from 100 permutations was 0.30. The correlation was significant for 72 out of the 100 permutations (two-tailed binomial *p* < 0.05). This suggests that our stimuli can index stable conflict effect differences between individuals.

Split-half reliability scores for the cognitive control scores were low to moderate: Stroop incongruent errors *r*_SB_ = 0.48, 95% CI [0.19,0.67], Flanker incongruent errors *r*_SB_ = 0.39, 95% CI [0.08,0.63] and AY errors *r*_SB_ = 0.66, 95% CI [0.47,0.80]. Task impurity and poor reliability are known issues with cognitive control measures. Possible reasons include changes in measurements within a participant due to greater familiarity with the task as the experiment progresses, fatigue, or even the adopting of different strategies on different trials (e.g. [57]). In cases of poor reliability, any correlations reported with an individual task are subject to interpretational difficulty.

Factor analysis is a commonly employed solution to this problem. Using multiple tasks that involve cognitive control, we can extract the part of the measurement that is most relevant and common across different tasks, separate from their unique sources of noise [41,57]. This improves construct validity. Further, we tested different participants in the proposed study than in the pilot and repeated the factor analysis, strengthening external validity.

The soundness of factor analyses results is evaluated by measures of communality (cf*.* reliability [58]). Communality refers to the proportion of variance for a variable that is explained by the common factor. In the pilot study, all six variables had higher communality than the recommended threshold of 0.2 [59]. Reading partial span (0.60), operation partial span (0.43), backwards digit span (0.34), Flanker Incongruent error (0.29), AY error (0.50), Stroop incongruent error (0.40)). For the proposed study, we adopted the same approach—reporting split-half reliabilities and communalities and removing any variable with communality less than 0.2.

### 2.7. Power analysis

We used the pilot data to conduct a power analysis (‘mixedpower’ function from ‘mixedpower’ package, v. 0.1.0 [60]) in R, which calculates power for mixed effects regression models as described by Kumle *et al*. [61]. The power (1 − *β*) was set at 0.80 and the significance level (*α*) at 0.05, two-tailed (please see appendix B for the model equation). For Hypothesis 2, we would require sample sizes up to *N* = 100 to detect a possible three-way interaction between individuals' cognitive control scores, current sentence trial type, and the previous Stroop type at Word2. Based on this, we decided on a final sample size of 104 (greater than 100 and a multiple of 8, for eight lists). We expected some exclusions due to performance on the reading and operation span tasks as in the pilot experiment.

## 3. Results

The pre-registered experiment was conducted using the same procedures as the pilot experiment (see the approved Stage 1 protocol here: https://osf.io/zcm5p/?view_only=5a763e14da25418e99f585f356bfbf7e). All data and scripts can be found here: https://osf.io/7kwqf/?view_only=7e24eb3857f0419ea3010585f65296e8.

### 3.1. Hypothesis 1: significant conflict modulation effect in the disambiguating region for SS and possibly PA

For the first hypothesis, we analysed reading times in the disambiguating region for each conflict type (SS and PA) separately. One hundred and forty-five adults participated. As anticipated from the pilot, we had some exclusions. Specifically, 41 participants were excluded because their comprehension accuracy on the sentence trials was below chance (less than 66/112) or they did not meet the criteria on the reading and operation span tasks. Data from the remaining participants (*N* = 104; 51 female) were analysed using the same procedure as with the pilot data. Less than 1% of the trials were excluded for having abnormally low or high RTs or outliers. Reading times were adjusted for length as described above. None of the participants had taken part in the pilot experiment, so this was an independent dataset.

For SS sentences, we tested mixed-effects models of length-adjusted reading times at the disambiguating verb (Word0) plus the following two words (Word1 and Word2). The model contained fixed effects of previous Stroop trial type, current sentence trial type, their interaction, trial number and random intercepts for participants and items (see appendix B; we could not fit models containing random slopes due to convergence error). The analyses were carried out for all trials (i.e. irrespective of accuracy on the previous Stroop trial) and for trials where participants responded accurately on the previous Stroop trial (i.e. Stroop correct only). Results revealed no significant interaction between the previous Stroop and current sentence trial type for all models (all trials—Word0: estimate = 0.01, s.e. = 0.03, *p* = 0.80; Word1: estimate = 0.04, s.e. = 0.03, *p* = 0.24; Word2: estimate = 0.01, s.e. = 0.03, *p* = 0.78; Stroop correct only—Word0: estimate = 0.01, s.e. = 0.03, *p* = 0.84; Word1: estimate = 0.03, s.e. = 0.03, *p* = 0.27; Word2: estimate = 0.01, s.e. = 0.03, *p* = 0.74). Appendix I shows the full model results.

For PA sentences, the analyses were focused on the length-adjusted reading times for the disambiguating preposition (Word0) plus the following two words (Word1 and Word2). We did not find a significant interaction between the previous Stroop trial type and the current sentence trial for any of the models (all trials—Word0: estimate = 0.04, s.e. = 0.02, *p* = 0.12; Word1: estimate = 0.004, s.e. = 0.03, *p* = 0.90; Word2: estimate = 0.03, s.e. = 0.03, *p* = 0.33; Stroop correct only—Word0: estimate = 0.04, s.e. = 0.02, *p* = 0.09; Word1: estimate = 0.0004, s.e. = 0.03, *p* = 0.99; Word2: estimate = 0.03, s.e. = 0.03, *p* = 0.35). See appendix J.

As in the pilot experiment, Hypothesis 1 was not supported by the data. In Bayes factors analyses, we found strong to very strong evidence in favour of H0 compared with H1 for both SS (Word0: 52.03 times more in favour of H0 compared with H1, Word1: 25.8 times, Word2: 51.6 times) and PA (Word0: 14.6 times more in favour of H0 compared with H1, Word1: 52.9 times, Word2: 32.6 times) conflict types.

### 3.2. Hypothesis 2: significant effect of individual differences in cognitive control on online processing for incongruent SS and potentially incongruent PA sentences

One additional participant was excluded due to greater than 90% errors on both congruent and incongruent Flanker trials, indicating that they did not understand or pay attention to the task. All subsequent analyses for Hypotheses 2 and 3 contained 103 participants (50 female).

**Figure 2.**
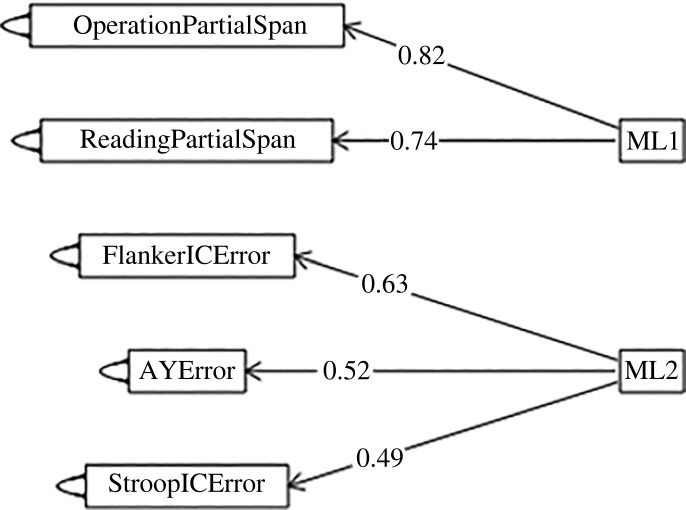
Path diagram from exploratory factor analysis (maximum likelihood with a varimax rotation). ML1 = working memory, ML2 = cognitive control.

In the factor analysis, backwards digit span did not pass the communality threshold of 0.2 and was therefore dropped. The other variables—errors for AY, Stroop incongruent and Flanker incongruent trials, and partial reading and operation span scores—passed the threshold. Kaiser–Meyer–Olkin (KMO) values for all of these variables were above 0.5 and the KMO measure was 0.6, indicating that the data were sufficient for EFA. Bartlett's test of sphericity revealed that there were patterned relationships between variables (*p* < 0.001). Using an eigenvalue cut-off of 1.0 and by looking at the scree plot, we retained two factors (figure 2). Reading partial span and operation partial span loaded onto one factor (hereafter, working memory factor) and errors from the AY trials, Stroop incongruent trials, and Flanker incongruent trials loaded into another factor (hereafter, cognitive control factor).

For SS, we ran mixed-effects regression models for the three words (Word0, Word1 and Word2) with fixed effects of previous Stroop trial type, current sentence trial type, cognitive control, their interaction, working memory, trial number and random intercepts for participants and items (see appendix B; we could not fit models containing random slopes due to convergence error). For Hypothesis 2a, the effect of interest was a three-way interaction between previous Stroop by current sentence trial type by cognitive control. Similar to Hypothesis 1, we report the results from all trials and for trials following correct Stroop trials only. See appendix K for the full table of results. Results revealed no significant three-way interactions (all trials—Word0: estimate = 0.03, s.e. = 0.02, *p* = 0.17; Word1: estimate = −0.03, s.e. = 0.02, *p* = 0.21; Word2: estimate = 0.03, s.e. = 0.02, *p* = 0.27; Stroop correct only—Word0: estimate = 0.03, s.e. = 0.02, *p* = 0.16; Word1: estimate = −0.03, s.e. = 0.02, *p* = 0.23; Word2: estimate = 0.03, s.e. = 0.02, *p* = 0.24). For Hypothesis 2b, the effect of interest was a two-way interaction between current sentence trial type and cognitive control corresponding to a significant effect of cognitive control for incongruent but not congruent sentences. There were no significant effects at any word (all trials—Word0: estimate = −0.01, s.e. = 0.01, *p* = 0.63; Word1: estimate = 0.02, s.e. = 0.02, *p* = 0.16; Word2: estimate = −0.01, s.e. = 0.02, *p* = 0.45; Stroop correct only—Word0: estimate = −0.01, s.e. = 0.01, *p* = 0.42; Word1: estimate = 0.02, s.e. = 0.02, *p* = 0.26; Word2: estimate = −0.01, s.e. = 0.02, *p* = 0.40).

The corresponding analyses for the PA conflict type (see Appendix L) revealed no relevant three-way or two-way interactions (all *p*s > 0.05). However, there were some marginally significant results, which we describe further under Exploratory analyses.

Contrary to the pilot results, we did not find support for Hypothesis 2a or 2b. For SS, we did not find the trait-level or trait-by-state effects of cognitive control that we found in the pilot experiment.

### 3.3. Hypothesis 3: significant effect of individual differences in cognitive control on offline comprehension for both SS and PA conflict types

Logistic regression of offline comprehension accuracy did not reveal any significant interactions between cognitive control and current sentence trial type (*p*s > 0.2; see appendix M for SS and N for PA). The main effect of cognitive control was marginally significant for both sentence types (SS sentences: estimate = −0.17, s.e. = 0.10, *p* = 0.09; PA sentences: estimate = −0.17, s.e. = 0.10, *p* < 0.1).^7^ Individuals with better cognitive control (fewer errors) tended to have higher sentence comprehension accuracy but this effect was weaker than in the pilot experiment.

### 3.4. Reliability analysis

Raw RTs were used to compute the reliability scores for Hypotheses 1 and 2. For Hypothesis 1 (*N* = 104), the Spearman–Brown (SB) corrected split-half reliability scores for SS sentences were *r*_SB_ = 0.76, 95% CI [0.68,0.82] at Word0, *r*_SB_ = 0.67, 95% CI [0.57,0.76] at Word1 and *r*_SB_ = 0.73, 95% CI [0.63,0.81] at Word2, and for PA sentences were *r*_SB_ = 0.66, 95% CI [0.55,0.76] at Word0, *r*_SB_ = 0.71, 95% CI [0.61,0.80] at Word1 and *r*_SB_ = 0.71, 95% CI [0.61,0.79] at Word2. For Hypothesis 2 (*N* = 103), split-half reliability scores for SS sentences were *r*_SB_ = 0.76, 95% CI [0.68,0.83] at Word0, *r*_SB_ = 0.68, 95% CI [0.57,0.77] at Word1 and *r*_SB_ = 0.73, 95% CI [0.63,0.81] at Word2, and for PA sentences were *r*_SB_ = 0.66, 95% CI [0.54,0.76] at Word0, *r*_SB_ = 0.71, 95% CI [0.62,0.80] at Word1 and *r*_SB_ = 0.71, 95% CI [0.61,0.79] at Word2. For Hypothesis 3 (*N* = 103), the split-half reliability score for SS sentences was *r*_SB_ = 0.56, 95% CI [0.42,0.68] and for PA sentences was *r*_SB_ = 0.61, 95% CI [0.46,0.73]. In summary, the dependent variables showed acceptable to good levels of reliability (0.56–0.76).

Split-half reliability scores for the cognitive control scores (*N* = 103) were low to moderate, as expected from the pilot experiment: Stroop incongruent errors *r*_SB_ = 0.46, 95% CI [0.27,0.61], Flanker incongruent errors *r*_SB_ = 0.57, 95% CI [0.40,0.71] and AY errors *r*_SB_ = 0.61, 95% CI [0.48,0.71]. Therefore, as proposed, we used factor analysis and applied a communality threshold. The five variables included in the final analysis all had communality greater than 0.2 (reading partial span (0.54), operation partial span (0.67), Flanker incongruent error (0.41), AY error (0.27), Stroop incongruent error (0.24)).

### 3.5. Exploratory analyses

In laying out our hypotheses and predictions (table 1), we considered the possibility that the effects found for SS might potentially not extend to PA. The pilot results offered some support for this idea for Hypotheses 2a and 2b by finding significant three-way and two-way interactions for SS but not PA. However, in the completed study, we found no interactions for SS but some marginally significant interactions for PA. We explored the nature of these PA effects in order to clarify whether they were similar to or different from the pilot results for SS because this would bear on the question of whether the two conflict types have a qualitatively similar or dissimilar relationship to cognitive control.

For Hypothesis 2 for PA, there was a marginally significant three-way previous Stroop by current sentence trial type by cognitive control interaction at word0 and word1, and a marginally significant two-way current sentence trial type by cognitive control interaction at word1 (see appendix L, all trials). We further explored the marginally significant three-way interaction at Word0 and Word1 by analysing congruent and incongruent sentences separately. At Word0, for congruent sentences, there was a marginally significant trait-level correlation (all trials—estimate = −0.03, s.e. = 0.02, *p* = 0.08; Stroop correct only—estimate = −0.03, s.e. = 0.02, *p* = 0.09) and a significant trait-by-state interaction (all trials—estimate = 0.03, s.e. = 0.01, *p* = 0.05; Stroop correct only—estimate = 0.03, s.e. = 0.01, *p* = 0.04). The trait-by-state interaction was the result of a marginally significant correlation with trait-level cognitive control when congruent sentences followed a congruent Stroop (all trials—estimate = −0.03, s.e. = 0.01, *p* = 0.06; Stroop correct only—estimate = −0.03, s.e. = 0.01, *p* = 0.07) but not an incongruent Stroop (all trials—*p* = 0.98; Stroop correct only—*p* = 0.86) trial. For incongruent sentences at Word0, there was no significant trait-level correlation (all trials—estimate = −0.01, s.e. = 0.02, *p* = 0.54; Stroop correct only—estimate = −0.01, s.e. = 0.02, *p* = 0.53) and no trait-by-state interaction (all trials—estimate = −0.01, s.e. = 0.02, *p* = 0.62; Stroop correct only—estimate = −0.003, s.e. = 0.02, *p* = 0.86). At Word1, for congruent sentences, there was a marginally significant trait-level correlation (all trials—estimate = −0.03, s.e. = 0.02, *p* = 0.08; Stroop correct only—estimate = −0.03, s.e. = 0.02, *p* = 0.10) but no trait-by-state interaction (all trials—estimate = 0.02, s.e. = 0.02, *p* = 0.17; Stroop correct only—estimate = 0.02, s.e. = 0.02, *p* = 0.13). For incongruent sentences at Word1, there was no significant trait-level correlation (all trials—estimate = −0.002, s.e. = 0.02, *p* = 0.92; Stroop correct only—estimate = −0.002, s.e. = 0.02, *p* = 0.90) and no trait-by-state interaction (all trials—estimate = −0.02, s.e. = 0.02, *p* = 0.23; Stroop correct only—estimate = −0.02, s.e. = 0.02, *p* = 0.32). To summarize, the marginal effects detected at Word0 and Word1 for PA resulted from a correlation with cognitive control for congruent but not incongruent sentences. Unlike the pilot results for SS, these do not reflect conflict-specific effects, which should affect incongruent sentences in particular. Thus, across two separate datasets, we found no support for individual differences in cognitive control impacting the processing of incongruent PA sentences.

## 4. Discussion

This study used a conflict modulation paradigm to investigate whether cognitive control modulated the processing of conflict during sentence comprehension, and whether the recruitment of cognitive control varied systematically according to type of sentential conflict, individual differences in cognitive control and task demands. We completed a pilot experiment to validate our approach, conduct power analyses and pre-register the experimental protocol. Subsequently, we conducted the planned research using the pre-registered protocol to collect an independent dataset and test three hypotheses. We discuss each hypothesis in turn below.

The first hypothesis pertained to whether the effects of cognitive control on sentence processing are observable at the group level for one or both of two types of sentential conflict—namely, syntax-semantics (SS) and phrase-attachment (PA) conflict. Neither the pilot nor the pre-registered experiment offered support for this hypothesis. We did not observe any effects of cognitive control on the processing of sentences containing either type of conflict. Bayes factors offered strong to very strong support for the null hypothesis. These null effects stand in contrast to prior demonstrations of conflict modulation within sentence processing [15–17]. Those studies were conducted in a laboratory setting while the current study collected data using a web-based platform. Participants in the web-based platform provided demographic information, including English language status, independently from the experiments they participated in. We also excluded participants based on accuracy in tasks involving English language stimuli. Therefore, we do not think that a difference in English language proficiency or attention to the tasks is the reason behind the discrepant results.

One alternative possibility is that the web-based studies contained a more diverse sample than the college student sample in university-based studies. This could impact the ability to detect group-based effects. The different results could also be due to differences in methodology. In this context, it is worth noting that prior positive evidence comes largely from the visual-world paradigm where participants selected pictures or moved pictures between locations after listening to sentences. By contrast, the current study used self-paced reading and did not involve any overt action. Thus, an open question raised by the present null results is whether conflict modulation is especially robust when language comprehension is embedded within the context of deciding between actions. Another possible reason could be differences in the stimuli. We normed the sentences and found that they induced the expected conflict effects, but we did not observe a significant effect of sentence type (incongruent versus congruent) in the actual conflict adaptation experiment. Therefore, it is possible that the incongruent sentences were not difficult enough to show a robust conflict modulation effect. Finally, although our studies used comparable inter-stimulus intervals as in other conflict modulation studies, the nature of the task (self-paced reading) may have led to longer and/or variable temporal intervals between the previous Stroop trial and the disambiguating region of the sentence, which in turn could have affected the chances of finding conflict modulation. Additional studies are needed to fully determine the generalizability of conflict modulation during sentence processing to different task contexts and stimuli (see also [18,19]).

Prior to conducting the study, we envisioned the possibility of not finding a group-level conflict modulation effect for a different reason, namely variability across individuals. Hypothesis 2 investigated this possibility by testing whether individual differences in cognitive control impacted conflict modulation. We tested for trait-level correlations and/or trait-by-state interactions to clarify if individuals with better cognitive control were better able to resolve conflict when reading incongruent sentences and/or mobilize cognitive control for that purpose when it is triggered by a previous Stroop trial. For PA sentences, neither the pilot nor the pre-registered experiment found relevant significant results. For SS sentences, we found mixed results in the pilot versus the pre-registered experiment. In the pilot experiment, we found support for both trait-level and trait-by-state relationships with cognitive control. However, in the pre-registered experiment, these results did not replicate. To better assess the contradictory evidence, we computed Bayes factors for the three-way interaction in the pilot and the pre-registered results. For the pilot experiment, the Bayes factors were 0.04, 0.04 and 0.28 at words 0, 1 and 2 respectively. For the pre-registered experiment, the Bayes factors were 0.05, 0.04 and 0.04. Thus, while the Bayes factor for word2 in the pilot experiment (0.28) could be interpreted as supporting neither H0 nor H1 strongly, the bulk of the evidence seems to favour the null hypothesis (20–25 times more likely). The lack of robust effects for SS, paralleling the null effects for PA, neither supports nor refutes the possibility of a difference between the two conflict types. Using the visual-world paradigm to test both sentence types *within the same participants* might be a useful future avenue for testing whether the two types show similar or different effects in a task context that has yielded detectable conflict modulation effects in previous studies [11,16].

By contrast to the first two hypotheses, Hypothesis 3 investigated the connection between cognitive control and offline rather than online processing. We predicted that any task that requires making decisions between competing responses should benefit from cognitive control. We found some support for this hypothesis in both the pilot and the pre-registered experiment. There were marginally significant or significant correlations with cognitive control in both datasets for both sentence types. The findings are consistent with a broad role for cognitive control in enhancing task performance either via post-interpretive processes [36] or the handling of domain-general task demands [5].

Turning to the question of domain-generality, the debate in the field is primarily about whether domain-general cognitive control can modulate immediate and automatic linguistic processing as detected via online measures. At least one previous study has noted modulation of online sentence processing by a non-verbal cognitive control task (Flanker; [17]). The present study used a linguistic Stroop task to modulate cognitive control within the conflict adaptation paradigm. Therefore, it cannot bear one way or another on the question of how non-linguistic cognitive control might or might not affect online comprehension. The results for Hypothesis 3 suggest a relationship between accuracies on comprehension questions on the one hand and factor scores obtained from multiple cognitive control tasks on the other. The former measure clearly relies on linguistic processing but could also include non-linguistic components (e.g. choosing between motor response options). The latter measure (factor scores) was calculated from cognitive control tasks that contained linguistic stimuli (Stroop) and those that did not (Flanker). Thus, neither of the two variables involved in the correlation is likely to be purely linguistic. A relationship between them does not violate assumptions about domain-specificity or domain-generality.

To summarize, we investigated if cognitive control recruitment for language processing varied systematically according to three factors—type of sentential ambiguity or conflict, individual differences in cognitive control, and task demands. The findings did not offer positive support for the first two factors. We did not find reliable group-level conflict modulation for either SS or PA conflict and also did not find robust individual differences effects for either conflict type. The results offer tentative support for the task demand hypothesis because we found marginally significant or significant correlations between cognitive control and offline comprehension accuracy but not online measures. When sentence comprehension was evaluated using an overt decision between response options, those with better cognitive control showed better accuracy. Future research can shed light on whether such task demands are experimental task-related artefacts that are extraneous to naturalistic comprehension or whether they share similarities with real-life contexts that also involve actions and tasks. Specifically, it would be interesting to know whether sentence comprehension that is embedded in the context of a naturalistic action (e.g. speaking to an interlocutor or responding to an interlocutor's request) is more likely to be modulated by cognitive control.

## Data Availability

All data and scripts are available on OSF: https://osf.io/zcm5p/?view_only=5a763e14da25418e99f585f356bfbf7e.

## Footnotes

1 Note that sentences like (1) describe scenarios that are unexpected given world knowledge, but they are not ungrammatical. Thus, they differ from animacy violations (e.g. *The meal was devouring the boy*; Kim & Osterhout [22]). Animacy is a grammatical factor encompassed by syntax under many linguistic theories. We chose stimuli involving semantic aspects that were clearly distinct from syntax because our hypotheses consider the possibility that cognitive control effects might vary for different types of sentences, which involve the need to reconcile independent processing streams versus not.

2 For gender assigned at birth, the split was 51 female and 53 male.

3 We use the term 'incongruent' for these PA sentences solely to be consistent with the corresponding labels for SS sentences [16]. Congruent, as used here, is synonymous with no-conflict and Incongruent, with conflict.

4 Because we planned to use exploratory factor analysis to extract variance shared between cognitive control tasks, we chose a task that has been shown to load onto a common factor with Stroop [38]. The AX-continuous performance test (AX-CPT) task tests both proactive and reactive cognitive control. Only reactive cognitive control is relevant to the present study. We used the same reactive control measure as Brothers *et al.*

5 Box-Cox test over the RTs (using the ‘boxcox’ function from the MASS package, v. 7.3-57. [34]) revealed a lambda value of -0.26. Log-transform is appropriate in this case, in addition to being the most commonly used transformation for positive values like RTs (e.g. James *et al.* [49]. See also: https://statmodeling.stat.columbia.edu/2019/08/21/you-should-usually-log-transform-your-positive-data/).

6 This procedure uses the unit information prior, which is a data-dependent prior with the mean at the maximum likelihood estimate and variance equal to the information from one observation.

7 Splitting by sentence trial type, for SS congruent sentences, there was a marginally significant correlation with cognitive control (estimate = −0.17, s.e. = 0.10, *p* = 0.09). For SS incongruent sentences, the correlation was significant (estimate = −0.20, s.e. = 0.07, *p* = 0.007). Similarly, for PA, the effect was marginally significant for congruent sentences (estimate = −0.18, s.e. = 0.10, *p* = 0.08) and significant for incongruent sentences (estimate = −0.31, s.e. = 0.08, *p* = 0.0001). In all cases, better cognitive control (lower errors) was associated with better comprehension accuracy (see appendices O and P for the full results).

8 We included trial number in all models to account for variation in performance as the experiment progresses (e.g. participants could get faster due to more practice with the task, or slower due to fatigue).

## Appendix A

**Table:** Syntax-semantics sentence stimuli.

congruent	incongruent
After the bankruptcy the company was **audited by the** determined tax agent.	During tax season the IRS employee was **audited by the** determined accountant.
During the sting operation the mobsters were **handcuffed by the** diligent detective.	After the drug raid the cops were **handcuffed by the** diligent anti-corruption squad.
After the tax error the accountant was **fired by the** frustrated shop owner.	After a failed presentation the supervisor was **fired by the** frustrated CEO.
Following orientation the recruit was **trained by the** nice policewoman.	Before the Superbowl the coach was **trained by the** nice public relations manager.
After being pulled over the driver was **breathalyzed by the** concerned state patrolman.	At the DUI check-point the traffic cop was **breathalyzed by the** concerned colleague.
During the university job fair the students were **recruited by the** competitive tech companies.	Prior to the baseball season the talent scouts were **recruited by the** competitive teams.
After the bombing the suspects were **interrogated by the** intimidating chief of police.	At the police station the detectives were **interrogated by the** intimidating commission.
At the checkout counter the shopper was **overcharged by the** shifty store worker.	At the store the cashier was **overcharged by the** shifty salesperson.
After years of embezzling the crook was **arrested by the** methodical FBI agent.	During the protest the policeman was **arrested by the** methodical inspector.
While eating lunch outside the celebrity was **photographed by the** pushy paparazzi.	On the red carpet the cameraman was **photographed by the** pushy tabloid reporter.
During the filming the actor was **directed by the** creative producer.	During the first rehearsal the conductor was **directed by the** creative musician.
During the party the new student was **bullied by the** popular girls.	At the school yard the jock was **bullied by the** popular football star.
From the airport the arriving passengers were **transported by the** licensed cab driver.	In the large aircraft the pilots were **transported by the** licensed captains.
After the exam the students were **graded by the** qualified teaching assistant.	During the finals week the professors were **graded by the** qualified peers.
On the talk show the guest was **teased by the** famous comic.	At the Oscars the host was **teased by the** famous celebrity.
While in office the mayor was **bribed by the** ruthless businessman.	To hide the felony the criminal was **bribed by the** ruthless politician.
After the classroom prank the freshman was **lectured by the** school principal.	During the class the teacher's aide was **lectured by the** school administrator.
While walking to the courtroom the criminal was **questioned by the** loud reporter.	At the press conference the journalist was **questioned by the** loud man.
During the field trip the teenagers were **chaperoned by the** strict parents.	At the high school dance the teachers were **chaperoned by the** strict principal.
Before entering the nightclub the frat boys were **scanned by the** distrustful bouncers.	At airport security the TSA agents were **scanned by the** distrustful managers.
At the mental health hospital the client was **analyzed by the** sympathetic psychologist.	During the therapy session the psychiatrist was **analyzed by the** sympathetic counselor.
When boarding the plane the elderly passenger was **escorted by the** friendly flight attendant.	At the film premiere the bodyguard was **escorted by the** friendly security team.
For the official portrait the queen was **painted by the** talented artist.	In the studio the artist was **painted by the** talented students.
At the chess tournament the prodigy was **coached by the** retired grand master.	At the gym the trainer was **coached by the** retired champion.
In the hostage crisis the civilians were **abducted by the** covert militia.	When the large UFO landed the aliens were **abducted by the** covert government agency.
In the sketchy neighborhood the pedestrian was **assaulted by the** devious gangster.	In the dark alley the thug was **assaulted by the** devious patrolman.
Before the marathon the athlete was **massaged by the** skilled physical therapist.	At the luxurious spa the therapist was **massaged by the** skilled co-worker.
After a fight on the court the player was **ejected by the** concerned official.	After the bad call the referee was **ejected by the** concerned sports organization.

**Table:** Phrase-attachment critical sentence stimuli.

Incongruent sentences (congruent sentences had an extra word indicated within parentheses)
Working at the valet stand Colin said put the car (that's) in the entrance into the parking lot and drive slowly.
In the subway Paige said put the ticket (that's) on the counter into the machine and pass through the gate.
In the messy living room Adam said put the blanket (that's) on the futon into the dresser and vacuum the floor.
Working in the law office Jim said put the document (that's) in the filing cabinet onto the clipboard and then sign it.
Working at the Italian restaurant Melissa said put the pasta (that's) in the pan onto the serving platter and sprinkle cheese on top.
In the living room Miranda said put the mug (that's) on the end table onto the coaster and turn on the TV.
In the master bedroom Ian said put the pillow (that's) on your bed into the closet and wash your sheets.
Working at the job fair Connor said put the pamphlet (that's) on the table into the folder and sign the paper.
Working at the take-out restaurant Stephanie said put the rice (that's) in the wok into the take out container and bag it up.
Working at the clothing store Mark said put the dress (that's) in the shop window into the shopping bag and ring them up.
Working in the cosmetics store Emma said put the lipstick (that's) on the makeup counter into the drawer and lock it.
In the entertainment room Sabrina said put the batteries (that are) in the remote into the trash can and take the garbage out.
Working at the salon Oscar said put the hair clip (that's) on the wig onto the vanity tray and get my scissors.
At the family picnic Kayla said put the plate (that's) on the napkin into the basket and fold the blanket.
In the office break room Katie said put the coffee powder (that's) in the espresso machine into the trash and get back to work.
In the gym Michelle said put the yoga mat (that's) on the floor onto the rack and cool down.
In the home theatre Alejandro said put the DVD (that's) in the case into the media player and turn the volume up.
In the bathroom Margaret said put the toothbrush (that's) in the holder into the recycling bin and get a new one.
At the apartment Ryan said put the chair (that's) in the balcony into the living room and close the door.
On the boat Diana said put the bait (that's) on your hook into the tackle box and try a different lure.
Working at the airport James said put the luggage (that's) on the conveyor belt into the plane and be gentle.
In the mansion Autumn said put the rug (that's) on the hardwood floor into the cupboard and start vacuuming.
Working in the pub Lucas said put the glass (that's) on the bar table into the dishwasher and turn it on.
In the kitchen Cassandra said put the banana (that's) in the pantry into the bowl and grab a knife.
Working at the loading dock Destiny said put the cargo (that's) in the truck onto the ship and depart.
Working at the tea shop Robert said put the cup (that's) on the saucer into the sink and wash the dishes.
At the taxicab stand Alexandria said put the suitcase (that's) in the trunk onto the cart and check the time.
At the burning building Brooke said put the ladder (that's) on the fire truck onto the wall and then climb it.

**Table:** Phrase-attachment filler sentence stimuli.

filler 1	filler 2
Working at the valet stand Colin said put the car in the entrance carefully.	Working at the valet stand Colin said put the car in the entrance for the customer.
In the messy living room Adam said put the blanket on the futon before you fluff the pillows.	In the messy living room Adam said put the blanket on the futon to make it look nice for our guest.
In the subway Paige said put the ticket on the counter before you go through the turnstile.	In the subway Paige said put the ticket on the counter to pay the fare.
Working at the Italian restaurant Melissa said put the pasta in the pan and then toss it with olive oil.	Working at the Italian restaurant Melissa said put the pasta in the pan with some butter.
In the living room Miranda said put the mug on the end table after you take a sip.	In the living room Miranda said put the mug on the end table and start the movie.
Working in the law office Jim said put the document in the filing cabinet and lock it.	Working in the law office Jim said put the document in the filing cabinet after getting the applicant's signature.
In the master bedroom Ian said put the pillow on your bed and fluff it.	In the master bedroom Ian said put the pillow on your bed and fold the sheets.
In the entertainment room Sabrina said put the batteries in the remote and make some popcorn.	In the entertainment room Sabrina said put the batteries in the remote because the old ones are dead.
Working at the job fair Connor said put the pamphlet on the table and display the brochures.	Working at the job fair Connor said put the pamphlet on the table before the event starts.
Working at the clothing store Mark said put the dress in the shop window and then help that customer.	Working at the clothing store Mark said put the dress in the shop window before we open.
Working at the take-out restaurant Stephanie said put the rice in the wok before you add the sauce.	Working at the take-out restaurant Stephanie said put the rice in the wok and then add some green onion.
At the family picnic Kayla said put the plate on the napkin and hand me the cheese.	At the family picnic Kayla said put the plate on the napkin and then bring out the wine.
Working at the salon Oscar said put the hairclip on the wig and add some hairspray.	Working at the salon Oscar said put the hairclip on the wig and wear it.
Working in the cosmetics store Emma said put the lipstick on the makeup counter after you help the customer.	Working in the cosmetics store Emma said put the lipstick on the makeup counter and arrange it by colour.
In the gym Michelle said put the yoga mat on the floor and lay down.	In the gym Michelle said put the yoga mat on the floor before you stretch.
In the bathroom Margaret said put the toothbrush in the holder and wash your hands.	In the bathroom Margaret said put the toothbrush in the holder to use later.
In the home theatre Alejandro said put the DVD in the case and turn off the TV.	In the home theatre Alejandro said put the DVD in the case so it's not damaged.
On the boat Diana said put the bait on your hook because the other bait won't work.	On the boat Diana said put the bait on your hook before you cast your line.
Working at the airport James said put the luggage on the conveyor belt for the arriving travellers.	Working at the airport James said put the luggage on the conveyor belt when it arrives.
In the office break room Katie said put the coffee powder in the espresso machine and grab the milk.	In the office break room Katie said put the coffee powder in the espresso machine before our shareholder meeting.
At the apartment Ryan said put the chair on the balcony before our guests arrive.	At the apartment Ryan said put the chair on the balcony while we clean up.
In the kitchen Cassandra said put the banana in the pantry when you put away the groceries.	In the kitchen Cassandra said put the banana in the pantry and cut some oranges.
Working at the tea shop Robert said put the cup on the saucer before you hand it to the customer.	Working at the tea shop Robert said put the cup on the saucer gently.
Working in the pub Lucas said put the glass on the bar table and charge the customer.	Working in the pub Lucas said put the glass on the bar table and then clean it.
In the mansion Autumn said put the rug on the hardwood floor to keep our feet warm.	In the mansion Autumn said put the rug on the hardwood floor for decoration.
At the burning building Brooke said put the ladder on the fire truck against the building.	At the burning building Brooke said put the ladder on the fire truck and bring the hose out.
Working at the loading dock Destiny said put the cargo in the truck before you take your trip.	Working at the loading dock Destiny said put the cargo in the truck quickly.
At the taxicab stand Alexandria said put the suitcase in the trunk and take me to the hotel.	At the taxicab stand Alexandria said put the suitcase in the trunk and get in the cab.

**Table:** Relative clause sentence stimuli.

Object relatives (subject relatives indicated within parentheses)
After the violent fight the campus security who the partiers hated (who hated the partiers) was handcuffing the fraternity members.
To find the culprit the detectives who the victims called (who called the victims) were interrogating the possible suspects.
After the financial scandal the tax agent who the accountants distrusted (who distrusted the accountants) was auditing the corrupt company.
At the job fair the HR manager who the attendees approached (who approached the attendees) was recruiting the promising undergrads.
After pulling over the car the patrolman who the passengers recognized (who recognized the passengers) was breathalyzing the drunk driver.
At the restaurant the head waiter who the customers appreciated (who appreciated the customers) was training the new hire.
For laundering corporate money the boss who the staff trusted (who trusted the staff) was firing the devious accountant.
Before the wedding started the cameraman who the groom knew (who knew the groom) was photographing the bridal party.
During the opera the conductor who the choir complimented (who complimented the choir) was directing the symphony orchestra.
During the long voyage the captain who the navy radioed (who radioed the navy) was transporting the tired migrants.
After the math final the lecturer who the students criticized (who criticized the students) was grading the exam papers.
Outside the school the jocks who the football coach liked (who liked the football coach) were bullying the shy kid.
During the riot the cops who the security helped (who helped the security) were arresting the angry protesters.
Outside the stadium the scalpers who the guards watched (who watched the guards) were overcharging the eager fans.
In the science lab the technicians who the reviewers contacted (who contacted the reviewers) were analyzing the biological samples.
At the TSA checkpoint the agents who the pilots knew (who knew the pilots) were scanning the travelers’ luggage.
At the local bar the crook who the politicians helped (who helped the politicians) was bribing the district attorney.
In the classroom the teacher who the principal liked (who liked the principal) was lecturing the bored students.
On the field trip the parents who the teachers supported (who supported the teachers) were chaperoning the middle schoolers.
During the famous trial the lawyer who the plaintiff emailed (who emailed the plaintiff) was questioning the lying defendant.
From the bleachers the fans who the cheerleaders applauded (who applauded the cheerleaders) were teasing the opponent players.
At the casino the senior employee who the management praised (who praised the management) was escorting the drunk gambler.
At the public playground the kidnapper who the local families knew (who knew the local families) was abducting the solitary child.
In the physical rehab centre the chiropractor who the coach contacted (who contacted the coach) was massaging the aching athlete.
To boost business sales the consultant who the workers observed (who observed the workers) was coaching the startup company.
For the art project the portrait artist who the critics disliked (who disliked the critics) was painting the beautiful model.
At the club the bouncer who the organizers assisted (who assisted the organizers) was ejecting the rowdy partiers.
At the prison the inmate who the guards despised (who despised the guards) was assaulting the corrections officer.

## Appendix B

**B.1. Mixed-effects model for Hypothesis 1**

lmer. Length-adjusted reading times Word0/1/2 ∼ 1 + Previous Stroop trial type * Current sentence trial type + Trial number + (1|Participants) + 1|Items)

Here and elsewhere, we have provided the maximal model that converged for the pilot data. We will include all random effects that converge. Trial number is a continuous variable and will be centred.

**B.2. Bayes factor calculation for Hypothesis 1**

full_lmer = lmer4::lmer(Length-adjusted reading times Word0/1/2 ∼ Previous Stroop trial type * Current sentence trial type + Trial number + 1|Participants) + (1|Items))

null_lmer = lmer4::lmer(Length-adjusted reading times Word0/1/2 ∼ Previous Stroop trial type + Current sentence trial type + Trial number + 1|Participants) + 1|Items))

Bayes factor in favour of null hypothesis = exp(BIC(full_lmer)-(null_lmer))/2)

**B.3. Mixed-effects model for Hypothesis 2**

lmer. Length-adjusted reading times Word0/1/2 ∼ 1 + Previous Stroop trial type * Current sentence trial type * Cognitive Control + Memory + Trial number + (1|Participants) + 1|Items)

Cognitive control factor scores, working memory factor scores, and trial number are continuous variables that will be centred.

**B.4. Logistic regression model for Hypothesis 3**

*g*lm. Comprehension accuracy ∼ 1 + Current sentence trial type * Cognitive Control + Memory + number, family = binomial

Cognitive control factor scores, working memory factor scores and trial number are continuous variables that will be centred. Note: if the model with random effects converges, we will use the glmer function instead of glm.

**B.5. Factor analysis model**

fa (data, nfactors = 2, fm = ’ml’, rotate = ’varimax’)

**B.6. Path diagram model equation obtained from the factor analysis**

fa.diagram (data, digits = 2, cut = 0.40, simple = F, errors = T)

**B.7. Power analysis model**

power = mixedpower (model = lmer. Length-adjusted reading times Word2, data, fixed_effects = c(‘Previous Stroop trial type’, ‘Current sentence trial type’, ‘Cognitive Control’, simvar = 'Participants’, steps = c(80,100,120), critical value = 2, n_sim = 1000)

## Appendix C

Pilot results for Hypothesis 1. Effects at the disambiguating verb (Word0) plus the following two words (Word1 and Word2) for syntax-semantics (SS) sentences. A previous Stroop by current sentence trial type interaction would indicate conflict modulation.

**Table:**

**all trials**				
**fixed effect**	**coef.**	**s.e.**	***t***	***p***
**Word0**				
intercept	0.10	0.02	5.11	<0.001***
previous Stroop trial incongruent^a^	−0.001	0.02	−0.03	0.97
current sentence trial incongruent^b^	0.01	0.02	0.43	0.67
interaction of previous Stroop and current sentence				
Stroop incongruent^a^ × sentence incongruent^b^	0.01	0.03	0.45	0.66
trial number	−0.001	0.0001	−12.91	<0.001***
random effect	s^2^			
participants	0.01			
items	0.003			
**Word1**				
intercept	0.10	0.03	3.65	<0.001***
previous Stroop trial incongruent^a^	−0.004	0.02	−0.17	0.87
current sentence trial incongruent^b^	0.004	0.02	0.16	0.88
interaction of previous Stroop and current sentence				
Stroop incongruent^a^ × sentence incongruent^b^	0.05	0.03	1.39	0.17
trial number	−0.002	0.0001	−13.17	<0.001***
random effect	s^2^			
participants	0.03			
items	0.003			
**Word2**				
intercept	0.13	0.02	6.21	<0.001***
previous Stroop trial incongruent^a^	0.01	0.02	0.34	.74
current sentence trial incongruent^b^	0.01	0.02	0.32	.75
interaction of previous Stroop and current sentence				
Stroop incongruent^a^ × sentence incongruent^b^	0.02	0.03	0.64	0.53
trial number	−0.002	0.0001	−12.81	<0.001***
random effect	s^2^			
participants	0.02			
items	0.003			
**Stroop correct only**				
**Word0**				
intercept	0.10	0.02	5.05	<0.001***
previous Stroop trial incongruent^a^	−0.001	0.02	−0.06	0.95
current sentence trial incongruent^b^	0.01	0.02	0.44	0.66
interaction of previous Stroop and current sentence				
Stroop incongruent^a^ × sentence incongruent^b^	0.02	0.03	0.52	0.61
trial number	−0.001	0.0001	−12.68	<0.001***
random effect	s^2^			
participants	0.01			
items	0.004			
**Word1**				
intercept	0.09	0.03	3.58	<0.001***
previous Stroop trial incongruent^a^	−0.005	0.02	−0.22	0.83
current sentence trial incongruent^b^	0.005	0.02	0.23	0.82
interaction of previous Stroop and current sentence				
Stroop incongruent^a^ × sentence incongruent^b^	0.05	0.03	1.40	0.17
trial number	−0.002	0.0001	−13.06	<0.001***
random effect	s^2^			
participants	0.03			
items	0.003			
**Word2**				
intercept	0.13	0.02	6.15	<0.001***
previous Stroop trial incongruent^a^	0.01	0.02	0.41	.68
current sentence trial incongruent^b^	0.01	0.02	0.31	0.75
interaction of previous Stroop and current sentence				
Stroop incongruent^a^ × sentence incongruent^b^	0.02	0.03	0.66	0.51
trial number	−0.002	0.0001	−12.66	<0.001***
random effect	s^2^			
participants	0.02			
items	0.003			

Note. Excluding the intercepts, coef. = model estimation of the change in reading times for each fixed effect; s.e. = standard error of the estimate; *t* = Satterthwaite's method, two-tailed; s^2^ = variance for by-participants random intercepts and by-items random intercepts.

^a^Reference is previous Stroop congruent trial.

^b^Reference is current sentence congruent trial.

## Appendix D

Pilot results for Hypothesis 1. Effects at the disambiguating preposition (Word0) plus the following two words (Word1 and Word2) for phrase-attachment (PA) sentences. A previous Stroop by current sentence trial type interaction would indicate conflict modulation.

**Table:**

**all trials**				
**fixed effect**	**coef.**	**s.e.**	***t***	***p***
**Word0**				
intercept	0.07	0.02	3.51	<0.001***
previous Stroop trial incongruent^a^	−0.01	0.02	−0.66	0.51
current sentence trial incongruent^b^	−0.03	0.02	−1.31	0.19
interaction of previous Stroop and current sentence				
Stroop incongruent^a^ × sentence incongruent^b^	0.02	0.03	0.61	0.54
trial number	−0.001	0.0001	−13.32	<0.001***
random effect	s^2^			
participants	0.02			
items	0.002			
**Word1**				
intercept	0.12	0.02	4.92	<0.001***
previous Stroop trial incongruent^a^	0.003	0.02	0.16	0.87
current sentence trial incongruent^b^	−0.004	0.02	−0.19	0.85
interaction of previous Stroop and current sentence				
Stroop incongruent^a^ × sentence incongruent^b^	−0.02	0.03	−0.54	0.59
trial number	−0.001	0.0001	−10.95	<0.001***
random effect	s^2^			
participants	0.03			
items	0.001			
**Word2**				
intercept	0.06	0.02	3.19	0.002**
previous Stroop trial incongruent^a^	−0.01	0.02	−0.34	0.73
current sentence trial incongruent^b^	0.04	0.02	1.75	0.08
interaction of previous Stroop and current sentence				
Stroop incongruent^a^ × sentence incongruent^b^	0.002	0.03	0.06	0.96
trial number	−0.001	0.0001	−11.61	<0.001***
random effect	s^2^			
participants	0.01			
items	0.003			
**Stroop correct only**				
**Word0**				
intercept	0.07	0.02	3.53	<0.001***
previous Stroop trial incongruent^a^	−0.01	0.02	−0.64	0.52
current sentence trial incongruent^b^	−0.02	0.02	−1.21	0.23
interaction of previous Stroop and current sentence				
Stroop incongruent^a^ × sentence incongruent^b^	0.02	0.03	0.55	0.58
trial number	−0.001	0.0001	−13.24	<0.001***
random effect	s^2^			
participants	0.02			
items	0.002			
**Word1**				
intercept	0.12	0.02	4.95	<0.001***
previous Stroop trial incongruent^a^	0.006	0.02	0.26	0.80
current sentence trial incongruent^b^	−0.004	0.02	−0.21	0.84
interaction of previous Stroop and current sentence				
Stroop incongruent^a^ × sentence incongruent^b^	−0.02	0.03	−0.61	0.55
trial number	−0.001	0.0001	−10.75	<0.001***
random effect	s^2^			
participants	0.03			
items	0.001			
**Word2**				
intercept	0.06	0.02	3.17	0.002**
previous Stroop trial incongruent^a^	−0.01	0.02	−0.42	0.68
current sentence trial incongruent^b^	0.04	0.02	1.70	0.09
interaction of previous Stroop and current sentence				
Stroop incongruent^a^ × sentence incongruent^b^	0.001	0.03	0.02	0.99
trial number	−0.001	0.0001	−11.13	<0.001***
random effect	s^2^			
participants	0.01			
items	0.003			

Note. Excluding the intercepts, coef. = model estimation of the change in reading times for each fixed effect; s.e. = standard error of the estimate; *t* = Satterthwaite's method, two-tailed; s^2^ = variance for by-participants random intercepts and by-items random intercepts.

^a^Reference is previous Stroop congruent trial.

^b^Reference is current sentence congruent trial.

## Appendix E

Pilot results for Hypothesis 2. Effects at the disambiguating verb (Word0) plus the following two words (Word1 and Word2) for syntax-semantics (SS) sentences. A three-way interaction between previous Stroop type, current sentence type and cognitive control would indicate different trait-level and trait-by-state interaction effects for incongruent versus congruent sentences.

**Table:**

all trials				
fixed effect	coef.	s.e.	*t*	*p*
**Word0**				
intercept	0.10	0.02	4.45	<0.001***
previous Stroop trial incongruent^a^	−0.003	0.02	−0.12	0.90
current sentence trial incongruent^b^	−0.02	0.02	−0.81	0.42
cognitive control	−0.02	0.02	−1.24	0.22
interaction of previous Stroop and current sentence				
Stroop incongruent^a^ × sentence incongruent^b^	0.04	0.03	1.27	0.21
interaction of previous Stroop and cognitive control				
Stroop incongruent^a^ × cognitive control	−0.01	0.02	−0.88	0.38
interaction of current sentence and cognitive control				
sentence incongruent^b^ × cognitive control	−0.02	0.02	−0.98	0.33
interaction of previous Stroop, current sentence and cognitive control				
Stroop incongruent^a^ × sentence incongruent^b^ × cognitive control	0.02	0.02	1.04	0.30
working memory	−0.002	0.02	−0.14	0.89
trial number	−0.001	0.0001	−12.72	<0.001***
random effect	s^2^			
participants	0.01			
items	0.003			
**Word1**				
intercept	0.06	0.02	2.62	0.01*
previous Stroop trial incongruent^a^	−0.01	0.02	−0.27	0.79
current sentence trial incongruent^b^	−0.02	0.02	−0.92	0.36
cognitive control	−0.02	0.02	−1.12	0.27
interaction of previous Stroop and current sentence				
Stroop incongruent^a^ × sentence incongruent^b^	0.05	0.03	1.53	0.13
interaction of previous Stroop and cognitive control				
Stroop incongruent^a^ × cognitive control	0.001	0.02	0.03	.98
interaction of current sentence and cognitive control				
sentence incongruent^b^ × cognitive control	−0.01	0.02	−0.66	.51
interaction of previous Stroop, current sentence and cognitive control				
Stroop incongruent^a^ × sentence incongruent^b^ × cognitive control	−0.02	0.03	−0.92	0.36
working memory	0.02	0.02	1.28	0.21
trial number	−0.002	0.0001	−12.17	<0.001***
random effect	s^2^			
participants	0.02			
items	0.003			
**Word2**				
intercept	0.12	0.02	4.97	<0.001***
previous Stroop trial incongruent^a^	0.004	0.03	0.15	0.88
current sentence trial incongruent^b^	−0.01	0.03	−0.45	0.65
cognitive control	−0.01	0.02	−1.26	0.21
interaction of previous Stroop and current sentence				
Stroop incongruent^a^ × sentence incongruent^b^	0.02	0.04	0.59	0.56
interaction of previous Stroop and cognitive control				
Stroop incongruent^a^ × cognitive control	−0.02	0.02	−0.91	0.36
interaction of current sentence and cognitive control				
sentence incongruent^b^ × cognitive control	−0.03	0.02	−1.55	0.12
interaction of previous Stroop, current sentence and cognitive control				
Stroop incongruent^a^ × sentence incongruent^b^ × cognitive control	0.06	0.03	2.19	0.03*
working memory	0.01	0.02	0.38	0.70
trial number	−0.002	0.0001	−11.13	<0.001***
random effect	s^2^			
participants	0.01			
items	0.01			
**two-way interaction of cognitive control and previous Stroop trial at Word2 for congruent sentences**				
intercept	0.12	0.02	5.24	<0.001***
previous Stroop trial incongruent^a^	0.01	0.03	0.21	0.84
cognitive control	−0.03	0.02	−1.35	0.18
interaction of previous Stroop and cognitive control				
Stroop incongruent^a^ × cognitive control	−0.02	0.02	−0.86	0.39
working memory	0.004	0.02	0.24	0.81
trial number	−0.001	0.0002	−7.72	<0.001***
random effect	s^2^			
participants	0.01			
items	0.004			
**two-way interaction of cognitive control and previous Stroop trial at Word2 for incongruent sentences**				
intercept	0.11	0.03	4.40	<0.001***
previous Stroop trial incongruent^a^	0.03	0.03	0.98	0.33
cognitive control	−0.05	0.02	−2.54	0.01*
interaction of previous Stroop and cognitive control				
Stroop incongruent^a^ × cognitive control	0.04	0.02	2.27	0.02*
working memory	0.01	0.02	0.44	0.66
trial number	−0.002	0.0002	−8.25	<0.001***
random effect	s^2^			
participants	0.02			
items	0.005			
**interaction at Word2 for incongruent sentences: effect of cognitive control when previous Stroop trial was congruent**				
intercept	0.11	0.03	4.34	<0.001***
cognitive control	−0.05	0.02	−2.78	0.008**
working memory	0.01	0.02	0.28	0.78
trial number	−0.002	0.0004	−4.58	<0.001***
random effect	s^2^			
participants	0.01			
items	0.01			
**interaction at Word2 for incongruent sentences: effect of cognitive control when previous Stroop trial was incongruent**				
intercept	0.13	0.02	5.41	<0.001***
cognitive control	−0.01	0.02	−0.52	0.61
working memory	0.01	0.02	0.46	0.65
trial number	−0.002	0.0002	−7.40	<0.001***
random effect	s^2^			
participants	0.02			
items	0.004			
**Stroop correct only**				
**Word0**				
intercept	0.10	0.02	4.39	<0.001***
previous Stroop trial incongruent^a^	−0.004	0.02	−0.16	0.88
current sentence trial incongruent^b^	−0.02	0.02	−0.66	0.51
cognitive control	−0.02	0.02	−1.22	0.23
interaction of previous Stroop and current sentence				
Stroop incongruent^a^ × sentence incongruent^b^	0.04	0.03	1.26	0.21
interaction of previous Stroop and cognitive control				
Stroop incongruent^b^ × cognitive control	−0.02	0.02	−1.01	0.31
interaction of current sentence and cognitive control				
sentence incongruent^b^ × cognitive control	−0.02	0.02	−1.01	0.31
interaction of previous Stroop, current sentence and cognitive control				
Stroop incongruent^a^ × sentence incongruent^b^ × cognitive control	0.03	0.02	1.14	0.26
working memory	−0.002	0.02	−0.13	0.89
trial number	−0.001	0.0001	−12.61	<0.001***
random effect	s^2^			
participants	0.01			
items	0.003			
**Word1**				
intercept	0.06	0.02	2.62	0.01*
previous Stroop trial incongruent^a^	−0.01	0.02	−0.43	0.67
current sentence trial incongruent^b^	−0.02	0.02	−0.82	0.41
cognitive control	−0.02	0.02	−1.12	0.27
interaction of previous Stroop and current sentence				
Stroop incongruent^a^ × sentence incongruent^b^	0.05	0.03	1.53	0.13
interaction of previous Stroop and cognitive control				
Stroop incongruent^a^ × cognitive control	0.001	0.02	0.06	0.95
interaction of current sentence and cognitive control				
sentence incongruent^b^ × cognitive control	−0.01	0.02	−0.70	0.49
interaction of previous Stroop, current sentence and cognitive control				
Stroop incongruent^a^ × sentence incongruent^b^ × cognitive control	−0.02	0.03	−0.85	0.39
working memory	0.02	0.02	1.26	0.21
trial number	−0.002	0.0001	−12.25	<0.001***
random effect	s^2^			
participants	0.02			
items	0.003			
**Word2**				
intercept	0.12	0.02	4.90	<0.001***
previous Stroop trial incongruent^a^	0.01	0.03	0.23	0.82
current sentence trial incongruent^b^	−0.01	0.03	−0.34	0.73
cognitive control	−0.03	0.02	−1.24	0.22
interaction of previous Stroop and current sentence				
Stroop incongruent^a^ × sentence incongruent^b^	0.02	0.04	0.51	0.61
interaction of previous Stroop and cognitive control				
Stroop incongruent^a^ × cognitive control	−0.02	0.02	−1.09	0.28
interaction of current sentence and cognitive control				
sentence incongruent^b^ × cognitive control	−0.03	0.02	−1.52	0.13
interaction of previous Stroop, current sentence and cognitive control				
Stroop incongruent^a^ × sentence incongruent^b^ × cognitive control	0.06	0.03	2.29	0.02*
working memory	0.01	0.02	0.41	0.69
trial number	−0.002	0.0001	−11.01	<0.001***
random effect	s^2^			
participants	0.01			
items	0.01			
**two-way interaction of cognitive control and previous Stroop trial at Word2 for congruent sentences**				
intercept	0.12	0.02	5.17	<0.001***
previous Stroop trial incongruent^a^	0.01	0.03	0.28	0.78
cognitive control	−0.03	0.02	−1.33	0.19
interaction of previous Stroop and cognitive control				
Stroop incongruent^a^ × cognitive control	−0.02	0.02	−1.01	0.31
working memory	0.004	0.02	0.24	0.81
trial number	−0.001	0.0002	−7.64	<0.001***
random effect	s^2^			
participants	0.01			
items	0.004			
**two-way interaction of cognitive control and previous Stroop trial at Word2 for incongruent sentences**				
intercept	0.11	0.03	4.46	<0.001***
previous Stroop trial incongruent^a^	0.02	0.03	0.95	0.35
cognitive control	−0.05	0.02	−2.51	0.01*
interaction of previous Stroop and cognitive control				
Stroop incongruent^a^ × cognitive control	0.04	0.02	2.25	0.02*
working memory	0.01	0.02	0.46	0.65
trial number	−0.002	0.0002	−8.20	<0.001***
random effect	s^2^			
participants	0.01			
items	0.005			
**interaction at Word2 for incongruent sentences: effect of cognitive control when previous Stroop trial was congruent**				
intercept	0.11	0.03	4.41	<0.001***
cognitive control	−0.05	0.02	−2.76	0.008**
working memory	0.01	0.02	0.31	0.76
trial number	−0.002	0.0004	−4.55	<0.001***
random effect	s^2^			
participants	0.01			
items	0.01			
**interaction at Word2 for incongruent sentences: effect of cognitive control when previous Stroop trial was incongruent**				
intercept	0.14	0.02	5.43	<0.001***
cognitive control	−0.01	0.02	−0.51	0.61
working memory	0.01	0.02	0.48	0.63
trial number	−0.002	0.0002	−7.33	<0.001***
random effect	s^2^			
participants	0.02			
items	0.004			

Note. Excluding the intercepts, coef. = model estimation of the change in reading times for each fixed effect; s.e. = standard error of the estimate; *t* = Satterthwaite's method, two-tailed; s^2^ = variance for by-participant random intercepts and by-items random intercepts.

^a^Reference is previous Stroop congruent trial.

^b^Reference is current sentence congruent trial.

## Appendix F

Pilot results for Hypothesis 2. Effects at the disambiguating verb (Word0) plus the following two words (Word1 and Word2) for phrase-attachment (PA) sentences. A three-way interaction between previous Stroop type, current sentence type and cognitive control would indicate different trait-level and trait-by-state interaction effects for incongruent versus congruent sentences.

**Table:**

**all trials**				
**fixed effect**	**coef****.**	**s.e.**	***t***	***p***
**Word0**				
intercept	0.07	0.02	3.20	0.002**
previous Stroop trial incongruent^a^	−0.01	0.02	−0.42	0.68
current sentence trial incongruent^b^	−0.03	0.02	−1.51	0.13
cognitive control	−0.03	0.02	−1.22	0.22
interaction of previous Stroop and current sentence				
Stroop incongruent^a^ × sentence incongruent^b^	0.01	0.03	0.47	0.64
interaction of previous Stroop and cognitive control				
Stroop incongruent^a^ × cognitive control	−0.01	0.02	−0.68	0.50
interaction of current sentence and cognitive control				
sentence incongruent^b^ × cognitive control	0.004	0.02	0.20	0.84
interaction of previous Stroop, current sentence and cognitive control				
Stroop incongruent^a^ × sentence incongruent^b^ × cognitive control	−0.001	0.02	−0.03	0.98
working memory	0.02	0.02	1.01	0.31
trial number	−0.001	0.0001	−12.30	<0.001***
random effect	s^2^			
participants	0.01			
items	0.002			
**Word1**				
intercept	0.12	0.03	4.15	0.01*
previous Stroop trial incongruent^a^	0.002	0.02	0.08	0.94
current sentence trial incongruent^b^	−0.02	0.02	−0.71	0.48
cognitive control	−0.05	0.03	−1.82	0.07
interaction of previous Stroop and current sentence				
Stroop incongruent^a^ × sentence incongruent^b^	−0.02	0.03	−0.61	0.54
interaction of previous Stroop and cognitive control				
Stroop incongruent^a^ × cognitive control	0.02	0.02	0.93	0.36
interaction of current sentence and cognitive control				
sentence incongruent^b^ × cognitive control	0.04	0.02	1.79	0.07
interaction of previous Stroop, current sentence and cognitive control				
Stroop incongruent^a^ × sentence incongruent^b^ × cognitive control	−0.05	0.03	−1.55	0.12
working memory	0.02	0.02	0.76	0.45
trial number	−0.001	0.0001	−9.23	<0.001***
random effect	s^2^			
participants	0.03			
items	0.001			
**Word2**				
intercept	0.07	0.02	2.92	0.004**
previous Stroop trial incongruent^a^	−0.01	0.03	−0.19	0.85
current sentence trial incongruent^b^	0.02	0.02	0.95	0.34
cognitive control	0.02	0.02	1.05	0.29
interaction of previous Stroop and current sentence				
Stroop incongruent^a^ × sentence incongruent^b^	0.01	0.04	0.28	0.78
interaction of previous Stroop and cognitive control				
Stroop incongruent^a^ × cognitive control	0.01	0.02	0.32	0.75
interaction of current sentence and cognitive control				
sentence incongruent^b^ × cognitive control	−0.02	0.02	−1.08	0.28
interaction of previous Stroop, current sentence and cognitive control				
Stroop incongruent^a^ × sentence incongruent^b^ × cognitive control	−0.03	0.03	−0.99	0.32
working memory	−0.01	0.02	−0.90	0.37
trial number	−0.002	0.0001	−11.29	<0.001***
random effect	s^2^			
participants	0.01			
items	0.002			
**Stroop correct only**				
**Word0**				
intercept	0.07	0.02	3.19	0.002**
previous Stroop trial incongruent^a^	−0.01	0.02	−0.46	0.65
current sentence trial incongruent^b^	−0.03	0.02	−1.54	0.13
cognitive control	−0.03	0.02	−1.20	0.23
interaction of previous Stroop and current sentence				
Stroop incongruent^a^ × sentence incongruent^b^	0.02	0.03	0.59	0.56
interaction of previous Stroop and cognitive control				
Stroop incongruent^a^ × cognitive control	−0.02	0.02	−1.01	0.31
interaction of current sentence and cognitive control				
sentence incongruent^b^ × cognitive control	0.004	0.02	0.20	0.84
interaction of previous Stroop, current sentence and cognitive control				
Stroop incongruent^a^ × sentence incongruent^b^ × cognitive control	0.01	0.02	0.23	0.82
working memory	0.02	0.02	1.003	0.31
trial number	−0.001	0.0001	−12.43	<0.001***
random effect	s^2^			
participants	0.01			
items	0.002			
**Word1**				
intercept	0.12	0.03	4.14	<0.001***
previous Stroop trial incongruent^a^	0.003	0.02	0.12	0.90
current sentence trial incongruent^b^	−0.02	0.02	−0.71	0.48
cognitive control	−0.05	0.03	−1.83	0.07
interaction of previous Stroop and current sentence				
Stroop incongruent^a^ × sentence incongruent^b^	−0.02	0.03	−0.66	0.51
interaction of previous Stroop and cognitive control				
Stroop incongruent^a^ × cognitive control	0.02	0.02	1.09	0.28
interaction of current sentence and cognitive control				
sentence incongruent^b^ × cognitive control	0.04	0.02	1.81	0.07
interaction of previous Stroop, current sentence and cognitive control				
Stroop incongruent^a^ × sentence incongruent^b^ × cognitive control	−0.05	0.03	−1.57	0.12
working memory	0.02	0.02	0.73	0.47
trial number	−0.001	0.0001	−9.05	<0.001***
random effect	s^2^			
participants	0.03			
items	0.001			
**Word2**				
intercept	0.07	0.02	2.88	0.005**
previous Stroop trial incongruent^a^	−0.01	0.03	−0.23	0.82
current sentence trial incongruent^b^	0.02	0.03	0.94	0.35
cognitive control	0.02	0.02	1.08	0.28
interaction of previous Stroop and current sentence				
Stroop incongruent^a^ × sentence incongruent^b^	0.01	0.04	0.24	0.81
interaction of previous Stroop and cognitive control				
Stroop incongruent^a^ × cognitive control	0.01	0.02	0.31	0.76
interaction of current sentence and cognitive control				
sentence incongruent^b^ × cognitive control	−0.02	0.02	−1.08	0.28
interaction of previous Stroop, current sentence and cognitive control				
Stroop incongruent^a^ × sentence incongruent^b^ × cognitive control	−0.03	0.03	−0.93	0.35
working memory	−0.01	0.02	−0.92	0.36
trial number	−0.002	0.0001	−10.92	<0.001***
random effect	s^2^			
participants	0.01			
items	0.003			

Note. Excluding the intercepts, coef. = model estimation of the change in reading times for each fixed effect; s.e. = standard error of the estimate; *t* = Satterthwaite's method, two-tailed; s^2^ = variance for by-participants random intercepts and by-items random intercepts.

^a^Reference is previous Stroop congruent trial.

^b^Reference is current sentence congruent trial.

## Appendix G

Pilot results for Hypothesis 3. Multiple logistic regression of sentence comprehension accuracy for syntax-semantics (SS) sentences. An effect of cognitive control would indicate a trait-level correlation for accuracy on both congruent and incongruent sentences, and an interaction between sentence type and cognitive control would indicate differential effects for congruent versus incongruent sentences.

**Table:**

offline comprehension accuracy				
fixed effect	coef.	s.e.	*Z*	*p*
intercept	2.93	0.16	17.54	<0.001***
sentence incongruent^a^	−0.69	0.21	−3.29	0.001**
cognitive control	−0.27	0.13	−2.10	0.04*
interaction of sentence trial and cognitive control				
sentence incongruent^a^ × cognitive control	0.005	0.16	0.03	0.97
working memory	0.13	0.09	1.53	0.13
trial number	0.002	0.001	1.17	0.24

Note. Excluding the intercepts, coef. = model estimation of the change in sentence comprehension accuracy (in log odds) from the reference category for each fixed effect; s.e. = standard error of the estimate; *Z* = value of the Wald Z test statistics, two-tailed.

^a^Reference is sentence congruent trial.

## Appendix H

Pilot results for Hypothesis 3. Multiple logistic regression of sentence comprehension accuracy for phrase-attachment (PA) sentences. An effect of cognitive control would indicate a trait-level correlation for accuracy on both congruent and incongruent sentences, and an interaction between sentence trial and cognitive control would indicate differential effects for congruent versus incongruent sentences.

**Table:**

offline comprehension accuracy				
fixed effect	coef.	s.e.	*Z*	*p*
intercept	3.47	0.22	15.51	<0.001***
sentence incongruent^a^	−0.83	0.27	−3.12	0.002**
cognitive control	−0.73	0.13	−5.53	<0.001***
interaction of sentence trial and cognitive control				
sentence incongruent^a^ × cognitive control	0.17	0.17	1.004	0.32
working memory	−0.12	0.09	−1.29	0.20
trial number	0.004	0.002	2.14	0.03*

Note. Excluding the intercepts, coef. = model estimation of the change in sentence comprehension accuracy (in log odds) from the reference category for each fixed effect; s.e. = standard error of the estimate; *Z* = value of the Wald Z test statistics, two-tailed.

^a^Reference is sentence congruent trial.

## Appendix I

Results for Hypothesis 1. Effects at the disambiguating verb (Word0) plus the following two words (Word1 and Word2) for syntax-semantics (SS) sentences. A previous Stroop by current sentence trial type interaction would indicate conflict modulation.

**Table:**

**all trials**				
**fixed effect**	**coef****.**	**s.e.**	***t***	***p***
**Word0**				
intercept	0.12	0.02	6.12	<0.001***
previous Stroop trial incongruent^a^	−0.01	0.02	−0.28	0.78
current sentence trial incongruent^b^	−0.01	0.02	−0.35	0.73
interaction of previous Stroop and current sentence				
Stroop incongruent^a^ × sentence incongruent^b^	0.01	0.03	0.25	0.80
trial number	−0.001	0.0001	−10.93	<0.001***
random effect	s^2^			
participants	0.02			
items	0.004			
**Word1**				
intercept	0.12	0.02	6.25	<0.001***
previous Stroop trial incongruent^a^	−0.03	0.02	−1.45	0.15
current sentence trial incongruent^b^	−0.01	0.02	−0.40	0.69
interaction of previous Stroop and current sentence				
Stroop incongruent^a^ × sentence incongruent^b^	0.04	0.03	1.19	0.24
trial number	−0.001	0.0001	−12.49	<0.001***
random effect	s^2^			
participants	0.02			
items	0.003			
**Word2**				
intercept	0.16	0.02	7.70	<0.001***
previous Stroop trial incongruent^a^	−0.02	0.02	−0.72	0.47
current sentence trial incongruent^b^	0.02	0.02	0.72	0.47
interaction of previous Stroop and current sentence				
Stroop incongruent^a^ × sentence incongruent^b^	0.01	0.03	0.28	0.78
trial number	−0.001	0.0001	−10.36	<0.001***
random effect	s^2^			
participants	0.02			
items	0.004			
**Stroop correct only**				
**Word0**				
intercept	0.12	0.02	6.15	<0.001***
previous Stroop trial incongruent^a^	−0.01	0.02	−0.25	0.81
current sentence trial incongruent^b^	−0.01	0.02	−0.35	0.73
interaction of previous Stroop and current sentence				
Stroop incongruent^a^ × sentence incongruent^b^	0.01	0.03	0.21	0.84
trial number	−0.001	0.0001	−11.10	<0.001***
random effect	s^2^			
participants	0.02			
items	0.004			
**Word1**				
intercept	0.12	0.02	6.16	<0.001***
previous Stroop trial incongruent^a^	−0.03	0.02	−1.34	0.18
current sentence trial incongruent^b^	−0.01	0.02	−0.37	0.71
interaction of previous Stroop and current sentence				
Stroop incongruent^a^ × sentence incongruent^b^	0.04	0.03	1.12	0.27
trial number	−0.001	0.0001	−12.62	<0.001***
random effect	s^2^			
participants	0.02			
items	0.003			
**Word2**				
intercept	0.16	0.02	7.71	<0.001***
previous Stroop trial incongruent^a^	−0.02	0.02	−0.76	0.45
current sentence trial incongruent^b^	0.02	0.02	0.70	0.49
interaction of previous Stroop and current sentence				
Stroop incongruent^a^ × sentence incongruent^b^	0.01	0.03	0.33	0.74
trial number	−0.001	0.0001	−10.47	<0.001***
random effect	s^2^			
participants	0.02			
items	0.004			

Note. Excluding the intercepts, coef. = model estimation of the change in reading times for each fixed effect; s.e. = standard error of the estimate; *t* = Satterthwaite's method, two-tailed; s^2^ = variance for by-participants random intercepts and by-items random intercepts.

^a^Reference is previous Stroop congruent trial.

^b^Reference is current sentence congruent trial.

## Appendix J

Results for Hypothesis 1. Effects at the disambiguating preposition (Word0) plus the following two words (Word1 and Word2) for phrase-attachment (PA) sentences. A previous Stroop by current sentence trial type interaction would indicate conflict modulation.

**Table:**

**all trials**				
**fixed effect**	**coef.**	**s.e.**	***t***	***p***
**Word0**				
intercept	0.08	0.02	5.11	<0.001***
previous Stroop trial incongruent^a^	−0.02	0.02	−0.94	0.35
current sentence trial incongruent^b^	−0.05	0.02	−3.25	0.002**
interaction of previous Stroop and current sentence				
stroop incongruent^a^ × sentence incongruent^b^	0.04	0.02	1.59	0.12
trial number	−0.001	0.0001	−13.54	<0.001***
random effect	s^2^			
participants	0.01			
items	0.001			
**Word1**				
intercept	0.13	0.02	6.51	<0.001***
previous Stroop trial incongruent^a^	0.005	0.02	0.24	0.81
current sentence trial incongruent^b^	−0.05	0.02	−2.38	0.02*
interaction of previous Stroop and current sentence				
Stroop incongruent^a^ × sentence incongruent^b^	0.004	0.03	0.13	0.90
trial number	−0.001	0.0001	−10.94	<0.001***
random effect	s^2^			
participants	0.02			
items	0.002			
**Word2**				
intercept	0.10	0.02	5.61	<0.001***
previous Stroop trial incongruent^a^	−0.02	0.02	−0.89	0.38
current sentence trial incongruent^b^	0.01	0.02	0.47	0.64
interaction of previous Stroop and current sentence				
Stroop incongruent^a^ × sentence incongruent^b^	0.03	0.03	0.97	0.33
trial number	−0.001	0.0001	−11.14	<0.001***
random effect	s^2^			
participants	0.01			
items	0.002			
**Stroop correct only**				
**Word0**				
intercept	0.08	0.02	5.12	<0.001***
previous Stroop trial incongruent^a^	−0.02	0.02	−1.00	0.32
current sentence trial incongruent^b^	−0.05	0.02	−3.22	0.002**
interaction of previous Stroop and current sentence				
Stroop incongruent^a^ × sentence incongruent^b^	0.04	0.02	1.72	0.09
trial number	−0.001	0.0001	−13.49	<0.001***
random effect	s^2^			
participants	0.01			
items	0.001			
**Word1**				
intercept	0.13	0.02	6.43	<0.001***
previous Stroop trial incongruent^a^	0.01	0.02	0.42	0.68
current sentence trial incongruent^b^	−0.05	0.02	−2.25	0.03*
interaction of previous Stroop and current sentence				
Stroop incongruent^a^ × sentence incongruent^b^	0.0004	0.03	0.02	0.99
trial number	−0.001	0.0001	−10.87	<0.001***
random effect	s^2^			
participants	0.02			
items	0.002			
**Word2**				
intercept	0.10	0.02	5.62	<0.001***
previous Stroop trial incongruent^a^	−0.02	0.02	−0.95	0.34
current sentence trial incongruent^b^	0.01	0.02	0.58	0.56
interaction of previous Stroop and current sentence				
Stroop incongruent^a^ × sentence incongruent^b^	0.02	0.03	0.93	0.35
trial number	−0.001	0.0001	−11.05	<0.001***
random effect	s^2^			
participants	0.01			
items	0.002			

Note. Excluding the intercepts, coef. = model estimation of the change in reading times for each fixed effect; s.e. = standard error of the estimate; *t* = Satterthwaite's method, two-tailed; s^2^ = variance for by-participants random intercepts and by-items random intercepts.

^a^Reference is previous Stroop congruent trial.

^b^Reference is current sentence congruent trial.

## Appendix K

Results for Hypothesis 2. Effects at the disambiguating verb (Word0) plus the following two words (Word1 and Word2) for syntax-semantics (SS) sentences. A three-way interaction between previous Stroop type, current sentence type and cognitive control would indicate different trait-level and trait-by-state interaction effects for incongruent versus congruent sentences.

**Table:**

**all trials**				
**fixed effect**	**coef.**	**s.e.**	***t***	***p***
**Word0**				
previous Stroop trial incongruent^a^	−0.01	0.02	−0.27	0.79
current sentence trial incongruent^b^	−0.01	0.02	−0.38	0.71
cognitive control	−0.01	0.02	−0.65	0.52
interaction of previous Stroop and current sentence				
stroop incongruent^a^ × sentence incongruent^b^	0.01	0.03	0.30	0.77
interaction of previous Stroop and cognitive control				
Stroop incongruent^a^ × cognitive control	−0.03	0.01	−1.93	0.054
interaction of current sentence and cognitive control				
sentence incongruent^b^ × cognitive control	−0.01	0.02	−0.49	0.63
interaction of previous Stroop, current sentence and cognitive control				
Stroop incongruent^a^ × sentence incongruent^b^ × cognitive control	0.03	0.02	1.39	0.17
working memory	−0.01	0.01	−0.86	0.39
trial number	−0.001	0.0001	−10.63	<0.001***
random effect	s^2^			
participants	0.02			
items	0.004			
**Word1**				
intercept	0.12	0.02	6.09	<0.001***
previous Stroop trial incongruent^a^	−0.03	0.02	−1.40	0.17
current sentence trial incongruent^b^	−0.01	0.02	−0.36	0.72
cognitive control	−0.01	0.02	−0.78	0.44
interaction of previous Stroop and current sentence				
Stroop incongruent^a^ × sentence incongruent^b^	0.04	0.03	1.17	0.25
interaction of previous Stroop and cognitive control				
Stroop incongruent^a^ × cognitive control	0.02	0.02	1.14	0.25
interaction of current sentence and cognitive control				
sentence incongruent^b^ × cognitive control	0.02	0.02	1.42	0.16
interaction of previous Stroop, current sentence and cognitive control				
Stroop incongruent^a^ × sentence incongruent^b^ × cognitive control	−0.03	0.02	−1.25	0.21
working memory	−0.01	0.01	−0.44	0.66
trial number	−0.001	0.0001	−12.15	<0.001***
random effect	s^2^			
participants	0.02			
items	0.003			
**Word2**				
intercept	0.16	0.02	7.58	<0.001***
previous Stroop trial incongruent^a^	−0.02	0.03	−0.72	0.47
current sentence trial incongruent^b^	0.02	0.02	0.72	0.48
cognitive control	0.01	0.02	0.45	0.65
interaction of previous Stroop and current sentence				
Stroop incongruent^a^ × sentence incongruent^b^	0.01	0.03	0.30	0.77
interaction of previous Stroop and cognitive control				
Stroop incongruent^a^ × cognitive control	−0.01	0.02	−0.52	0.61
interaction of current sentence and cognitive control				
sentence incongruent^b^ × cognitive control	−0.01	0.02	−0.75	0.45
interaction of previous Stroop, current sentence and cognitive control				
Stroop incongruent^a^ × sentence incongruent^b^ × cognitive control	0.03	0.02	1.11	0.27
working memory	0.002	0.02	0.17	0.87
trial number	−0.001	0.0001	−10.15	<0.001***
random effect	s^2^			
participants	0.02			
items	0.004			
**Stroop correct only**				
**Word0**				
intercept	0.12	0.02	6.09	<0.001***
previous Stroop trial incongruent^a^	−0.01	0.02	−0.25	0.80
current sentence trial incongruent^b^	−0.01	0.02	−0.37	0.71
cognitive control	−0.01	0.02	−0.71	0.48
interaction of previous Stroop and current sentence				
Stroop incongruent^a^ × sentence incongruent^b^	0.01	0.03	0.26	0.80
interaction of previous Stroop and cognitive control				
Stroop incongruent^a^ × cognitive control	−0.03	0.01	−1.94	0.053
interaction of current sentence and cognitive control				
sentence incongruent^b^ × cognitive control	−0.01	0.01	−0.81	0.42
interaction of previous Stroop, current sentence and cognitive control				
Stroop incongruent^a^ × sentence incongruent^b^ × cognitive control	0.03	0.02	1.41	0.16
working memory	−0.01	0.01	−0.86	0.39
trial number	−0.001	0.0001	−10.79	<0.001***
random effect	s^2^			
participants	0.02			
items	0.004			
**Word1**				
intercept	0.12	0.02	6.00	<0.001***
previous Stroop trial incongruent^a^	−0.03	0.02	−1.30	0.20
current sentence trial incongruent^b^	−0.01	0.02	−0.33	0.74
cognitive control	−0.01	0.02	−0.79	0.43
interaction of previous Stroop and current sentence				
Stroop incongruent^a^ × sentence incongruent^b^	0.03	0.03	1.10	0.27
interaction of previous Stroop and cognitive control				
Stroop incongruent^a^ × cognitive control	0.02	0.02	1.20	0.23
interaction of current sentence and cognitive control				
sentence incongruent^b^ × cognitive control	0.02	0.02	1.14	0.26
interaction of previous Stroop, current sentence and cognitive control				
Stroop incongruent^a^ × sentence incongruent^b^ × cognitive control	−0.03	0.02	1.19	0.23
working memory	−0.01	0.01	−0.50	0.62
trial number	−0.001	0.0001	−12.28	<0.001***
random effect	s^2^			
participants	0.02			
items	0.003			
**Word2**				
intercept	0.16	0.02	7.59	<0.001***
previous Stroop trial incongruent^a^	−0.02	0.03	−0.75	0.45
current sentence trial incongruent^b^	0.02	0.02	0.69	0.49
cognitive control	0.01	0.02	0.46	0.65
interaction of previous Stroop and current sentence				
Stroop incongruent^a^ × sentence incongruent^b^	0.01	0.03	0.35	0.73
interaction of previous Stroop and cognitive control				
Stroop incongruent^a^ × cognitive control	−0.01	0.02	−0.61	0.54
interaction of current sentence and cognitive control				
sentence incongruent^b^ × cognitive control	−0.01	0.02	−0.84	0.40
interaction of previous Stroop, current sentence and cognitive control				
Stroop incongruent^a^ × sentence incongruent^b^ × cognitive control	0.03	0.02	1.18	0.24
working memory	0.002	0.01	0.15	0.88
trial number	−0.001	0.0001	−10.25	<0.001***
random effect	s^2^			
participants	0.02			
items	0.004			

Note. Excluding the intercepts, coef. = model estimation of the change in reading times for each fixed effect; s.e. = standard error of the estimate; *t* = Satterthwaite's method, two-tailed; s^2^ = variance for by-participant random intercepts and by-items random intercepts.

^a^Reference is previous Stroop congruent trial.

^b^Reference is current sentence congruent trial.

## Appendix L

Results for Hypothesis 2. Effects at the disambiguating verb (Word0) plus the following two words (Word1 and Word2) for phrase-attachment (PA) sentences. A three-way interaction between previous Stroop type, current sentence type and cognitive control would indicate different trait-level and trait-by-state interaction effects for incongruent versus congruent sentences.

**Table:**

**all trials**				
**fixed effect**	**coef.**	**s.e.**	***t***	***p***
**Word0**				
intercept	0.08	0.02	5.06	<0.001***
previous Stroop trial incongruent^a^	−0.02	0.02	−1.06	0.29
current sentence trial incongruent^b^	−0.06	0.02	−3.39	0.001**
cognitive control	−0.03	0.02	−1.83	0.07
interaction of previous Stroop and current sentence				
Stroop incongruent^a^ × sentence incongruent^b^	0.04	0.02	1.77	0.08
interaction of previous Stroop and cognitive control				
Stroop incongruent^a^ × cognitive control	0.03	0.01	1.92	0.06
interaction of current sentence and cognitive control				
sentence incongruent^b^ × cognitive control	0.02	0.01	1.22	0.22
interaction of previous Stroop, current sentence and cognitive control				
Stroop incongruent^a^ × sentence incongruent^b^ × cognitive control	−0.03	0.02	−1.68	0.09
working memory	−0.002	0.01	−0.20	0.84
trial number	−0.001	0.0001	−13.23	<0.001***
random effect	s^2^			
participants	0.01			
items	0.001			
**Word1**				
intercept	0.13	0.02	6.45	<0.001***
previous Stroop trial incongruent^a^	0.004	0.02	0.20	0.85
current sentence trial incongruent^b^	−0.05	0.02	−2.40	0.02*
cognitive control	−0.03	0.02	−1.81	0.07
interaction of previous Stroop and current sentence				
Stroop incongruent^a^ × sentence incongruent^b^	0.004	0.03	0.14	0.89
interaction of previous Stroop and cognitive control				
Stroop incongruent^a^ × cognitive control	0.02	0.02	1.38	0.17
interaction of current sentence and cognitive control				
sentence incongruent^b^ × cognitive control	0.03	0.02	1.95	0.051
interaction of previous stroop, current sentence and cognitive control				
Stroop incongruent^a^ × sentence incongruent^b^ × cognitive control	−0.04	0.02	−1.82	0.07
working memory	0.003	0.02	0.20	0.85
trial number	−0.001	0.0001	−10.60	<0.001***
random effect	s^2^			
participants	0.02			
items	0.002			
**Word2**				
intercept	0.10	0.02	5.59	<0.001***
previous Stroop trial incongruent^a^	−0.02	0.02	−0.89	0.38
current sentence trial incongruent^b^	0.01	0.02	0.40	0.69
cognitive control	−0.002	0.02	−0.13	0.90
interaction of previous Stroop and current sentence				
Stroop incongruent^a^ × sentence incongruent^b^	0.03	0.03	1.02	0.31
interaction of previous Stroop and cognitive control				
Stroop incongruent^a^ × cognitive control	−0.003	0.02	−0.21	0.83
interaction of current sentence and cognitive control				
sentence incongruent^b^ × cognitive control	−0.01	0.02	−0.58	0.56
interaction of previous Stroop, current sentence and cognitive control				
Stroop incongruent^a^ × sentence incongruent^b^ × cognitive control	−0.002	0.02	−0.09	0.93
working memory	0.003	0.01	0.25	0.80
trial number	−0.001	0.0001	−10.83	<0.001***
random effect	s^2^			
participants	0.01			
items	0.002			
**Stroop correct only**				
**Word0**				
intercept	0.08	0.02	5.09	<0.001***
previous Stroop trial incongruent^a^	−0.02	0.02	−1.12	0.26
current sentence trial incongruent^b^	−0.06	0.02	−3.36	0.001**
cognitive control	−0.03	0.01	−1.78	0.08
interaction of previous Stroop and current sentence				
Stroop incongruent^a^ × sentence incongruent^b^	0.05	0.02	1.89	0.06
interaction of previous Stroop and cognitive control				
Stroop incongruent^a^ × cognitive control	0.03	0.01	2.02	0.04*
interaction of current sentence and cognitive control				
sentence incongruent^b^ × cognitive control	0.02	0.01	1.14	0.25
interaction of previous Stroop, current sentence and cognitive control				
Stroop incongruent^a^ × sentence incongruent^b^ × cognitive control	−0.03	0.02	−1.50	0.13
working memory	−0.003	0.01	−0.23	0.82
trial number	−0.001	0.0001	−13.22	<0.001***
random effect	s^2^			
participants	0.01			
items	0.001			
**Word1**				
intercept	0.13	0.02	6.37	<0.001***
previous Stroop trial incongruent^a^	0.01	0.02	0.36	0.72
current sentence trial incongruent^b^	−0.05	0.02	−2.28	0.03*
cognitive control	−0.03	0.02	−1.69	0.09
interaction of previous Stroop and current sentence				
Stroop incongruent^a^ × sentence incongruent^b^	0.001	0.03	0.02	0.98
interaction of previous Stroop and cognitive control				
Stroop incongruent^a^ × cognitive control	0.02	0.02	1.50	0.13
interaction of current sentence and cognitive control				
sentence incongruent^b^ × cognitive control	0.03	0.02	1.79	0.07
interaction of previous Stroop, current sentence and cognitive control				
Stroop incongruent^a^ × sentence incongruent^b^ × cognitive control	−0.04	0.02	−1.76	0.08
working memory	0.02	0.02	0.73	0.47
trial number	−0.001	0.0001	−9.05	<0.001***
random effect	s^2^			
participants	0.02			
items	0.002			
**Word2**				
intercept	0.10	0.02	5.59	<0.001***
previous Stroop trial incongruent^a^	−0.02	0.02	−0.96	0.34
current sentence trial incongruent^b^	0.01	0.02	0.51	0.61
cognitive control	−0.001	0.02	−0.08	0.94
interaction of previous Stroop and current sentence				
Stroop incongruent^a^ × sentence incongruent^b^	0.03	0.03	0.97	0.34
interaction of previous Stroop and cognitive control				
Stroop incongruent^a^ × cognitive control	−0.001	0.02	−0.04	0.97
interaction of current sentence and cognitive control				
sentence incongruent^b^ × cognitive control	−0.01	0.02	−0.66	0.51
interaction of previous Stroop, current sentence and cognitive control				
Stroop incongruent^a^ × sentence incongruent^b^ × cognitive control	−0.002	0.02	−0.07	0.94
working memory	0.002	0.01	0.18	0.85
trial number	−0.001	0.0001	−10.75	<0.001***
random effect	s^2^			
participants	0.01			
items	0.002			

Note. Excluding the intercepts, coef. = model estimation of the change in reading times for each fixed effect; s.e. = standard error of the estimate; *t* = Satterthwaite's method, two-tailed; s^2^ = variance for by-participants random intercepts and by-items random intercepts.

^a^Reference is previous Stroop congruent trial.

^b^Reference is current sentence congruent trial.

## Appendix M

Results for Hypothesis 3. Multiple logistic regression of sentence comprehension accuracy for syntax-semantics (SS) sentences. An effect of cognitive control would indicate a trait-level correlation for accuracy on both congruent and incongruent sentences and an interaction between sentence type and cognitive control would indicate differential effects for congruent versus incongruent sentences.

**Table:**

offline comprehension accuracy				
fixed effect	coef.	s.e.	*Z*	*p*
intercept	2.68	0.11	24.68	<0.001***
sentence incongruent^a^	−0.68	0.14	−4.98	<0.001***
cognitive control	−0.17	0.10	−1.71	0.09
interaction of sentence trial and cognitive control				
sentence incongruent^a^ × cognitive control	−0.03	0.12	−0.26	0.79
working memory	0.05	0.06	0.80	0.42
trial number	0.0001	0.001	0.10	0.92

Note. Excluding the intercepts, coef. = model estimation of the change in sentence comprehension accuracy (in log odds) from the reference category for each fixed effect; s.e. = standard error of the estimate; *Z* = value of the Wald Z test statistics, two-tailed.

^a^Reference is sentence congruent trial.

## Appendix N

Results for Hypothesis 3. Multiple logistic regression of sentence comprehension accuracy for phrase-attachment (PA) sentences. An effect of cognitive control would indicate a trait-level correlation for accuracy on both congruent and incongruent sentences and an interaction between sentence trial and cognitive control would indicate differential effects for congruent versus incongruent sentences.

**Table:**

offline comprehension accuracy				
fixed effect	coef.	s.e.	*Z*	*p*
intercept	2.88	0.12	24.09	<0.001***
sentence incongruent^a^	−0.47	0.15	−3.08	0.002**
cognitive control	−0.17	0.10	−1.67	0.10
interaction of sentence trial and cognitive control				
sentence incongruent^a^ × cognitive control	−0.14	0.13	−1.08	0.28
working memory	0.08	0.07	1.14	0.26
trial number	0.01	0.001	4.10	<0.001***

Note. Excluding the intercepts, coef. = model estimation of the change in sentence comprehension accuracy (in log odds) from the reference category for each fixed effect; s.e. = standard error of the estimate; *Z* = value of the Wald Z test statistics, two-tailed.

^a^Reference is sentence congruent trial.

## Appendix O

Exploratory analysis for Hypothesis 3. Multiple logistic regression of sentence comprehension accuracy for syntax-semantics (SS) congruent and incongruent sentences separately.

**Table:**

offline comprehension accuracy				
fixed effect	coef.	s.e.	*Z*	*p*
*congruent sentences*				
intercept	2.68	0.11	24.64	<0.001***
cognitive control	−0.17	0.10	−1.71	0.09
working memory	0.05	0.11	0.42	0.67
trial number	−0.002	0.001	−1.34	0.18
*incongruent sentences*				
intercept	2.00	0.08	24.30	<0.001***
cognitive control	−0.20	0.07	−2.68	0.007**
working memory	0.05	0.08	0.69	0.49
trial number	0.002	0.001	1.23	0.22

## Appendix P

Exploratory analysis for Hypothesis 3. Multiple logistic regression of sentence comprehension accuracy for phrase-attachment (PA) congruent and incongruent sentences separately.

**Table:**

offline comprehension accuracy				
fixed effect	coef.	s.e.	*Z*	*p*
*congruent sentences*				
intercept	2.94	0.13	22.73	<0.001***
cognitive control	−0.18	0.10	−1.72	0.08
working memory	0.21	0.10	2.02	0.04*
trial number	0.01	0.002	3.60	<0.001***
*incongruent sentences*				
intercept	2.40	0.10	24.24	<0.001***
cognitive control	−0.31	0.08	−3.84	<0.001***
working memory	−0.02	0.10	−0.22	0.83
trial number	0.004	0.002	2.19	0.03*
